# Unique Angiogenesis From Cardiac Arterioles During Pericardial Adhesion Formation

**DOI:** 10.3389/fcvm.2021.761591

**Published:** 2022-02-03

**Authors:** Kenji Namiguchi, Tomohisa Sakaue, Mikio Okazaki, Kaho Kanno, Yuhei Komoda, Fumiaki Shikata, Mie Kurata, Noritaka Ota, Yoshiaki Kubota, Hirotsugu Kurobe, Takashi Nishimura, Junya Masumoto, Shigeki Higashiyama, Hironori Izutani

**Affiliations:** ^1^Department of Cardiovascular and Thoracic Surgery, Ehime University Graduate School of Medicine, Toon, Japan; ^2^Department of Cell Growth and Tumor Regulation, Proteo-Science Center, Toon, Japan; ^3^Department of General Thoracic Surgery, Okayama University Graduate School of Medicine, Okayama, Japan; ^4^Department of Pathology, Division of Analytical Pathology, Ehime University Graduate School of Medicine, Toon, Japan; ^5^Department of Pathology, Proteo-Science Center, Toon, Japan; ^6^Department of Anatomy, Keio University School of Medicine, Tokyo, Japan; ^7^Department of Biochemistry and Molecular Genetics, Ehime University Graduate School of Medicine, Toon, Japan; ^8^Department of Molecular and Cellular Biology, Research Center, Osaka International Cancer Institute, Osaka, Japan

**Keywords:** angiogenesis, mouse model, pericardial adhesions, fibrosis, arteriole

## Abstract

**Objectives:**

The molecular mechanisms underlying post-operative pericardial adhesions remain poorly understood. We aimed to unveil the temporal molecular and cellular mechanisms underlying tissue dynamics during adhesion formation, including inflammation, angiogenesis, and fibrosis.

**Methods and Results:**

We visualized cell-based tissue dynamics during pericardial adhesion using histological evaluations. To determine the molecular mechanism, RNA-seq was performed. Chemical inhibitors were administered to confirm the molecular mechanism underlying adhesion formation. A high degree of adhesion formation was observed during the stages in which collagen production was promoted. Histological analyses showed that arterioles excessively sprouted from pericardial tissues after the accumulation of neutrophils on the heart surface in mice as well as humans. The combination of RNA-seq and histological analyses revealed that hyperproliferative endothelial and smooth muscle cells with dedifferentiation appeared in cytokine-exposed sprouting vessels and adhesion tissue but not in quiescent vessels in the heart. SMAD2/3 and ERK activation was observed in sprouting vessels. The simultaneous abrogation of PI3K/ERK or TGF-β/MMP9 signaling significantly decreased angiogenic sprouting, followed by inhibition of adhesion formation. Depleting MMP9-positive neutrophils shortened mice survival and decreased angiogenic sprouting and fibrosis in the adhesion. Our data suggest that TGF-β/matrix metalloproteinase-dependent tissue remodeling and PI3K/ERK signaling activation might contribute to unique angiogenesis with dedifferentiation of vascular smooth muscle cells from the contractile to the synthetic phenotype for fibrosis in the pericardial cavity.

**Conclusions:**

Our findings provide new insights in developing prevention strategies for pericardial adhesions by targeting the recruitment of vascular cells from heart tissues.

## Introduction

Pericardial adhesions are frequently recognized in repeat open-heart surgeries and complicate the procedure (1, 2). Growing evidence indicates that the risk of adverse events, such as intraoperative injury and bleeding, is increased during cardiac reoperations with adhesiotomy. Therefore, understanding the molecular mechanism underlying adhesion formation is necessary to prevent its occurrence (2).

The heart consists of various cell types, including cardiac myocytes, myofibroblasts, vascular smooth muscle cells (VSMCs), vascular endothelial cells, and mesothelial cells (3). Mesothelial cells typically surround myocardial tissues and maintain homeostasis of the pericardial cavity *via* serous fluid secretion (4). Of the various cell types in the pericardial cavity, mesothelial cells play a central role as the initiators of adhesion formation through multi-step reactions after pericardiotomy (5). Previous investigations using a bacterial toxin-induced pericardial adhesion model in sheep demonstrated that mesothelial cell transition from adhesion to free-floating type in the pericardial cavity induces inflammatory reactions (6–8). Following this, fibrin accumulation then occurs on the injured epicardial surface after the development of a scaffold for subsequent fibrosis (6). Finally, numerous inflammatory cells and fibroblasts invade the injured area, leading to collagen fiber production that eventually fills the pericardial space (7). Although new blood vessels have also been observed in collagen-deposited areas, the temporal cell behaviors of endothelial cells, myofibroblasts, and their source tissues during adhesion formation are poorly understood.

Blood vessels support the maintenance and survival of various tissues or organs by delivering nutrition and oxygen (9). Hypoxic and inflammatory signals induce the proliferation and migration of endothelial cells through activation of vascular endothelial growth factor (VEGF) receptors by ligands such as VEGF-A, -B, -C, and -D. The molecular mechanisms underlying the formation of new blood vessels in different developmental stages have been well characterized using a retinal angiogenesis murine model (10–13). Excessive leading and following cell proliferation of endothelial cells frequently occur during the growth and extension stages. Immature vascular trees are subsequently covered by pericytes, including myofibroblasts, for stabilization or specification into arteries (13), followed by the formation of lumen structures for blood perfusion. Nevertheless, the molecular and cellular mechanisms underlying pathological angiogenesis observed in adult cardiovascular diseases and repair are yet to be elucidated.

Molecular-based analyses of inflammatory signaling in pericardial fibrosis have been previously reported. Transforming growth factor-beta (TGF-β) is reportedly a central player in the activation of inflammatory reactions and the production of the extracellular matrix for fibrosis (14). The activation of TGF-β signaling also contributes to the transition from epithelial or endothelial cells to mesenchymal cells, promoting tissue fibrosis (15, 16). A previous human study reported elevated TGF-β levels in both serum and pericardial fluid of patients with pericarditis (17). Further, a pericardial adhesion model in pigs also demonstrated excessive upregulated expression of tumor necrosis factor-α, TGF-β, and phosphorylated SMAD, indicating that TGF-β is essential for inflammation in the pericardial cavity and adhesion formation (18). However, no studies have investigated the role of TGF-β in the early stages of pericardial adhesion.

We have previously established a simple model of pericardial adhesion in mice (19). In the current study, we conducted spatiotemporal visualization of histological changes using this model to elucidate the molecular and cellular mechanisms underlying tissue dynamics during adhesion formation, including inflammation, angiogenesis, and fibrosis.

## Methods and Materials

### Animals and Surgical Procedure

All animal experiments were conducted under the approval of the Ehime University Animal Care Committee (Project number: 05-RO-7-1 and 05-RO-42) and performed in accordance with the standards of the committee using the approved animal protocols. C57/Black6 (B6) mice were obtained from CLEA Japan (Tokyo, Japan). To generate endothelial cell-specific reporter mice, *Rosa26-EGFP* mice *(*The Jackson Laboratory, Bar Harbor, ME, Stock No. 012429) were crossed with *Cdh5*^*CreERT*2^ mice (11). To label the endothelial cells with an enhanced green fluorescent protein (EGFP), *Rosa26*^*EGFP*^:*Cdh5*^*CreERT*2^ mice were administered tamoxifen (catalog no. T5648., Sigma-Aldrich, St. Louis, MO, USA) *via* intraperitoneal injection (5 mg/40 g mouse body weight) for 3 consecutive days before talc administration. The induction of pericardial adhesion in mice was conducted as per a previously reported method (19). Briefly, all mice (male, 8 weeks) were anesthetized via mixed injection of ketamine (80 mg/kg) and xylazine (10 mg/kg). After laparotomy with minimal incision, 300 μl of a saline-based talc solution (2.5 mg/g) (Unitalc; Nobelpharma, Tokyo, Japan) was injected into the pericardial cavity via the diaphragm using a 27G needle. To establish the bleeding model, 300 μl of blood collected from donor mice was injected into the pericardial cavities of recipient mice (19). The chest cavities were excised and fixed with neutral buffered formalin. For histology and gene expression analyses, mice were euthanized by cervical dislocation under anesthesia. Adhesion severity was scored according to a previously described method (19), defined as follows: Grade 0, no adhesion; Grade 1, adhesion tissue was easily divided; Grade 2, adhesion tissue was partially divided; Grade 3, adhesion tissue adhered completely to the epicardium.

### Reagents and Antibodies

Hydrogen peroxide solution was obtained from Wako Pure Chemical Industries (Osaka, Japan). Anti-alpha smooth muscle actin (αSMA) mouse (catalog no. sc-56499) and anti-proliferating cell nuclear antigen (PCNA) rabbit (catalog no. sc-7907) antibodies were purchased from Santa Cruz Biotechnology, Inc. (Dallas, TX, USA). Anti-CD31 rat antibodies (catalog no. DIA-310) were obtained from Dianova (Hamburg, Germany). Anti-MMP9 rabbit (catalog no. ab38898), anti-TAGLN goat (catalog no. ab10135), anti-CD31 rabbit (catalog no. ab28364), and anti-CD34 mouse (catalog no. ab81289) antibodies were obtained from Abcam (Cambridge, UK). Anti-Phospho-p44/42 MAPK (ERK1/2) (Thr202/Tyr204) (catalog no. 9101) and anti-Phospho-Smad2 (Ser465/467) / Smad3 (Ser423/425) (D6G10) rabbit (catalog no.9510) antibodies were purchased from Cell Signaling Technology (Beverly, MA, USA). Anti-green fluorescent protein (GFP) rabbit antibodies (catalog no. 598) and anti-Glycoprotein M6a (GPM6A) monoclonal antibodies (catalog no. D055-3) were purchased from MBL Life Science, Inc. (Nagoya, Japan). Anti-myeloperoxidase (MPO) Antibodies (catalog no. AF3667) were obtained from R&D Systems (Minneapolis, MN, USA). Alexa Fluor^®^ 488 anti-mouse/human CD11b Antibodies (catalog no. 101219) and APC anti-mouse Ly-6G Antibody (catalog no. 127613) were obtained from BioLegend (San Diego, CA, USA). Anti-Ly6G/Ly6C (Gr-1) neutralizing antibodies (catalog no. BP0075) and Rat IgG2b Isotype control anti-KLH InVivoPlus antibodies (Clone: LTF-2) (catalog no. BP0090) were obtained from Bio X Cell, West Lebanon, NH). Anti-fibrinogen β chain (FGβ) rabbit antibodies (catalog no. HPA001900) were obtained from Atlas Antibodies, Inc. (Stockholm, Sweden). The Histofine^®^ Mouse Stain (peroxidase-conjugated anti-mouse IgG), Histofine^®^ Simple Stain™ Mouse MAX PO (anti-rabbit IgG), Histofine^®^ Simple Stain™ Mouse MAX PO (anti-rat IgG), and Histofine^®^ DAB substrate kits were obtained from Nichirei Bioscience Inc. (Tokyo, Japan). Alexa Fluor 488-conjugated anti-rat IgG (H+L) donkey (catalog no. A21208), anti-mouse IgG (H + L) goat (catalog no. A11001), and anti-rabbit IgG (H + L) goat (catalog no. A32731) antibodies, and Alexa Fluor 568-conjugated anti-rabbit IgG (H + L) goat (catalog no. A11036), and anti-mouse IgG (H + L) goat (catalog no. A11004) antibodies were purchased from Thermo Fisher Scientific (Waltham, MA, USA). Hoechst 33342 solution was purchased from Molecular Probes, Inc. (Eugene, OR, USA). ISOGEN II was obtained from Nippon Gene, Inc. (Tokyo, Japan). Gelatin Zymography Kit (Cosmo Bio type) (Catalog no. AK47) was obtained from Cosmo Bio Co., Ltd. (Tokyo, Japan). Monoclonal Anti-β-Actin antibody (catalog no. A5441), SB-431542 (catalog no. 616461), PD98059 (catalog no. P215), and LY294002 (catalog no. L9908) were obtained from Sigma (St. Louis, MO, USA). Marimastat (catalog no. S7156) was purchased from Selleckchem (Houston, TX, USA).

### Visualization of Tissue Structure and Fibrosis

Mouse chest cavities were excised and fixed using 10% neutral buffered formalin for 24 h. After decalcification with 10% ethylenediaminetetraacetic acid for 3 weeks, the tissues were embedded in paraffin and sliced into 5 μm sections. Paraffin-embedded heart tissue from the autopsy was used for comparative investigations of fibrosis and structure comparison between mice and humans. All clinical experiments were approved by the Ehime University Internal Review Board (protocol no.2105012), and informed consent was obtained based on the opt-out principle. Hematoxylin and eosin (H and E) and Masson's trichrome staining were performed to visualize tissue structures and collagen deposition, respectively. After the specimens were deparaffinized with xylene, samples were rehydrated and stained as previously described (19). For H and E staining, slides were stained with Meyer's hematoxylin solution for 15 min, rinsed with water for 10 min, and then stained with eosin, followed by washing with water. For Masson's trichrome staining, samples were treated with a mixture of 10% potassium dichromate and 10% trichloroacetic acid for 30 min, rinsed for 3 min, and then treated with Weigert's iron hematoxylin for 5 min. Samples were subsequently stained with 0.75% Orange G solution (Wako, Osaka, Japan) for 1 min and rinsed with acetic acid. Samples were then treated with xylidine ponceau acid for 20 min, 2.5% phosphotungstic acid for 10 min, and aniline blue for 5 min. Sagittal images of the chest cavity were obtained using a BZ-X800 microscope (Keyence, Kyoto, Japan). High-magnification images were acquired using a BX-51 Olympus microscope (Olympus, Tokyo, Japan).

### Immunostaining

To visualize the localization of FGB, anti-rabbit IgG HRP conjugate (Promega, Madison, WI. Catalog no. W402B. 1:4,000), αSMA, CD31, PCNA, MMP9, GPM6A phosphor-ERK, and phosphor-SMAD2/3, the tissues were deparaffinized with xylene. For antigen activation, the samples were heated for 15 min at 120°C in 10 mM sodium citrate buffer (pH 6.0) using an autoclave. Following inactivation of endogenous peroxidase with 3% hydrogen peroxide solution, samples were treated with blocking reagent Dako REAL^TM^ Antibody Diluent (Dako, Carpinteria, CA, USA) for 30 min. Anti-αSMA (1:2,000), CD31 (1:200), PCNA (1:1,000), MMP9 (1:1,000), GPM6A (1:400), phosphor-ERK (1:200), or phosho-SMAD2/3 (1:200) antibodies were probed onto the tissues and incubated overnight at 4°C. After washing with phosphate-buffered saline (PBS), the sections were treated with HRP-conjugated goat anti-mouse (for αSMA and PCNA), rat (for CD31 and GPM6A), and rabbit [for MMP9, phosphor-ERK, and phosho-SMAD2/3 (1:200)] antibodies (1:1,000) for 45 min at room temperature. The antigens were colored through treatment with the DAB substrate solution. After staining the nuclei with the hematoxylin solution, the sections were sealed with a cover glass. Tissue images were visualized as described above.

To investigate tissue co-localization, immunofluorescence assays were performed using multiple antibodies. After deparaffinization and antigen activation, samples were blocked using Dako REAL^TM^ Antibody Diluent, followed by reaction with the following primary antibody mixtures overnight at 4 °C: anti-αSMA (1:200)/CD31 (1:200), anti-αSMA (1:200)/TAGLN (1:200), anti-αSMA (1:200)/GFP (1:200), anti-αSMA (1:200)/PCNA (1:200), anti-CD31 (1:200)/PCNA (1:200), anti-MMP9 (1:500)/CD31 (1:200), anti-MMP9 (1:500)/CD31 (1:200), anti-MPO (1:400)/MMP9 (1/500), anti-CD34 (1:200)/CD31 (1:200), anti-CD34 (1:200)/αSMA (1:200), anti-pERK (1:200)/αSMA (1:200)/CD31 (1:200), or anti-pSMAD2/3 (1:200)/αSMA (1:200)/CD31 (1:200). After washing with PBS three times, the specimens were probed with the following fluorescent secondary antibody mixtures for 2 h at RT: Alexa Fluor 488-conjugated goat anti-rat IgG (1:1,000)/Alexa Fluor 568-conjugated goat anti-mouse IgG (1:1,000) for αSMA/CD31; Alexa Fluor 488-conjugated goat anti-rabbit IgG (1:1,000)/Alexa Fluor 568-conjugated goat anti-mouse IgG (1:1,000) for αSMA/GFP; Alexa Fluor 488-conjugated goat anti-mouse IgG (1:1,000)/Alexa Fluor 568-conjugated goat anti-rabbit IgG (1:1,000) for αSMA/PCNA and αSMA/CD34; Alexa Fluor 488-conjugated goat anti-rat IgG (1:1,000)/Alexa Fluor 568-conjugated goat anti-rabbit IgG (1:1,000) for CD31/PCNA, CD31/CD34, and MMP9/CD31; Alexa Fluor 488-conjugated donkey anti-rabbit IgG (1:1,000)/Alexa Fluor 568-conjugated donkey anti-goat IgG (1:1,000) for MMP9/MPO; Alexa Fluor 488-conjugated donkey anti-mouse IgG (1:1,000)/Alexa Fluor 568-conjugated donkey anti-goat IgG (1:1,000) for αSMA/TAGLN; and Alexa Fluor 488-conjugated goat anti-rat IgG (1:1,000)/Alexa Fluor 568-conjugated goat anti-rabbit IgG (1:1,000) for CD31/CD34. Following nuclear staining with Hoechst 33342, immunofluorescence signals were detected using an A1 confocal laser microscope (Nikon Co., Tokyo, Japan).

### RNA Isolation and Sequencing

Pericardial adhesion tissues and the myocardium were separately harvested from euthanized mice, homogenized with the ISOGEN II reagent, and stored at −20°C until further use. Total RNA was isolated according to the manufacturer's instructions for the ISOGEN II reagent. Purified total RNA was quantified using the NanoDrop 2000 spectrophotometer (Thermo Fisher Scientific), and RNA integrity score was evaluated using the Bioanalyzer RNA 6000 Nano Assay Kit (Agilent Technologies, Santa Clara, CA, USA). RNA sequencing using the MiSeq system (Illumina Inc., San Diego, Cam USA) was performed as previously described (20). Signal pathway and gene enrichment analyses were performed using DAVID version 6.8 bioinformatics software (http://david.abcc.ncifcrf.gov).

### Treatment With Chemical Inhibitors

The induction of pericardial adhesion in mice was conducted as described above. To investigate the functional roles of TGF-β/SMAD, PI3K/Akt, MEK/ERK, and MMP signaling during adhesion formation, TGF-β type1 receptor inhibitor (385 μg/kg, SB431542), MEK/ERK pathway inhibitor (2.7 mg/kg, PD98059), PI3K-kinase inhibitor (1.2 mg/kg, LY294002), MMPs inhibitor (25 mg/kg, Marimastat) were simultaneously injected into the pericardial cavity. To evaluate the inhibitory activity of TGF-β/SMAD/MMP signaling or PI3K/Akt/MEK/ERK signaling during adhesion formation, a combination of TGF-β type1 receptor inhibitor (385 μg/kg, SB431542) and MMPs inhibitor (25 mg/kg, Marimastat) or MEK/ERK pathway inhibitor (2.7 mg/kg, PD98059) and PI3K-kinase inhibitor (1.2 mg/kg, LY294002) was injected into the pericardial cavity. The chemical compounds were intraperitoneally administered on days 2 and 4. All compounds were dissolved with 5% DMSO in corn oil (Sigma-Aldrich, USA). Mice were euthanized on day 6, and the adhesion severity was scored as described above.

### Flow Cytometry

To quantify the cell population of neutrophils in the peripheral blood and heart tissue of adhesion-induced mice, Ly6g and CD11b double-positive cells were selectively collected using a flow cytometer. TALC-injected and sham mice were anesthetized *via* mixed injection of ketamine (80 mg/kg) and xylazine (10 mg/kg), and blood was collected using heparin-treated 23G needles. After perfusion with PBS, heart tissues were excised and digested with PBS-based collagenase (Worthington Biochemical, Freehold, NJ, USA) solution for 3 h at 37°C. After digestion, the samples were filtered through 40 μm cell strainers (BD Biosciences, Bedford, MA), and the cells were labeled with Alexa Fluor^®^ 488 anti-mouse/human CD11b antibodies and APC anti-mouse Ly-6G antibody. Peripheral blood treated with the antibodies were hemolyzed using OptiLyse C Lysing Solution according to the manufacturer's instructions. The samples were then washed with 10% fetal bovine serum and analyzed using a FACS Calibur flow cytometer (Becton Dickinson, San Jose, CA).

### Western Blotting

The heart tissues from talc-injected mice at each time point (day 0-7) were homogenized with Laemmli SDS sample buffer. After centrifugation, the protein concentration of the supernatant was measured using DC/RC Protein Assay Kit (Bio-Rad, Hercules, CA, USA), and 20 μg of the extracted protein was subjected to 10-20% gradient polyacrylamide gels (Oriental Instruments, Kanagawa, Japan). Proteins were transferred to the polyvinylidene fluoride membrane and probed with anti-MMP9, TAGLN, and β-actin antibodies, followed by treatment with anti-rabbit IgG HRP conjugate (Promega, Madison, WI. Catalog no. W402B. 1:4,000) for MMP9, anti-goat IgG HRP conjugate (Promega. Catalog no. V8051. 1:4,000) for TAGLN, and anti-mouse IgG HRP conjugate (Promega. Catalog no. W4021. 1:4,000) for β-actin. Chemiluminescent western blot images were obtained using a LAS-4000 (GE Healthcare Life Sciences, South Logan, UT, USA).

### Gelatin Zymography

The enzymatic activities of MMP9 were measured using a Gelatin Zymography Kit following the manufacturer's instructions. Forty micrograms of the extracted proteins from heart tissues were separated by SDS polyacrylamide gel electrophoresis, and the gel was incubated in the reaction buffer for 36 h at 37°C. The bands were visualized by staining with coomassie blue staining solution.

### Depletion of Ly6g-Positive Cells

To eliminate neutrophils, mice were injected intraperitoneally with 387 μg of Ly6g neutralizing antibodies or isotype control antibodies in 200 μl PBS, as previously reported (21). After 2 days, pericardial adhesions were induced by talc injections. Both antibodies were injected every 2 days. On the seventh day after talc injection, heart tissues were excised and adhesion scores measured, followed by histological analyses by Masson's trichrome staining and immunohistochemical staining for CD31 and αSMA.

### Statistical Analysis

All data are presented as mean ± standard error (SE). GraphPad Prism Ver. 7 (GraphPad Software, San Diego, CA, USA) was used to conduct a one-way analysis of variance (ANOVA) for normal distributions and the Kruskal-Wallis test for non-normal distributions. Statistical significances between two groups were calculated using Student's *t*-tests (parametric variables) and Mann-Whitney *U*-test (non-parametric variables). The normality of the obtained experimental data was assessed using the Shapiro-Wilk normality test. ImageJ software (NIH) was used for quantification histological data. ns.: not significant. ND.: not detected.

## Results

### Visualization of Tissue Dynamics During Adhesion Formation

**Figure 1:**
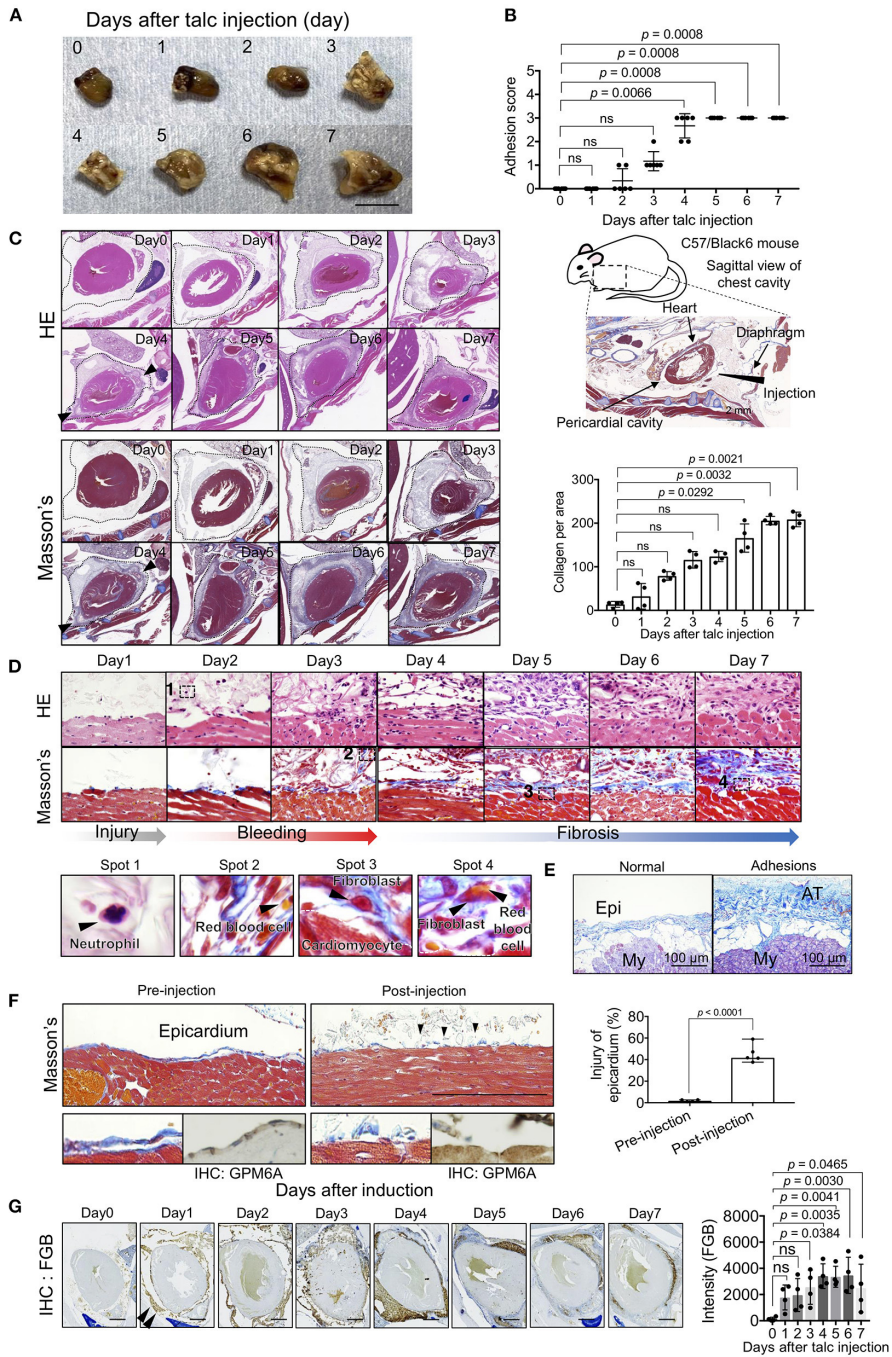
Spatiotemporal evaluation of pericardial adhesion formation. **(A)** Excised hearts within 7 days of adhesion induction (talc injection). Bar = 8 mm. **(B)** Adhesion scores of the excised heart tissues (*n* = 6). Data are presented as means ± SE. Statistical analyses were performed using Kruskal-Wallis test with Dunn's multiple comparison test (non-parametric variables). **(C)** H and E (left upper) and Masson's trichrome (left lower) staining of the sagittal sections of the chest cavity within 7 days of adhesion induction. The broken lines indicate the pericardium. Arrowheads indicate the points of cell invasion with fibrosis. The right upper panel shows a schematic image of the sagittal view of the mouse chest cavity surrounded by a broken line. Arrowheads and arrows in the right upper panel indicate the injection site and tissue descriptions, respectively. The right lower panel shows the quantified collagen deposits-stained blue in the pericardial cavity of the left lower panels (*n* = 4). Data are presented as means ± SE. Statistical analyses were performed using Kruskal-Wallis test with Dunn's multiple comparison test (non-parametric variables). **(D)** Temporal cell dynamics of myocardial tissues within 7 days of talc injection. The lower panels show magnified views of areas 1-4 in the upper panels. **(E)** Masson's trichrome-stained human heart tissues with or without adhesions. Bar = 100 μm. Epi, epicardium; My, myocardium; AT, adhesion tissue. **(F)** Masson's trichrome-stained epicardium, pre- (left) or post- (left) induction. Thoracic cavities were excised at the third hour after talc injection. Arrowheads indicate the injured epicardial layer after injection. Lower panels show magnified views of Masson's trichrome (left) and IHC for GPM6A (right). Right panel shows the quantitative analysis of epicardial injury in the pericardium (*n* = 5). Bar = 100 μm. Data are presented as means ± SE. Statistical analyses were performed using a two-tailed *t*-test (parametric variables). **(G)** Assessment of the bleeding process during pericardial adhesion formation via immunohistochemical staining using anti-fibrinogen β chain antibodies. Arrowheads indicate staining areas. The right panel shows the quantifications of stained signals of IHC for FGB in the pericardial cavity (*n* = 4). Data are presented as means ± SE. Statistical analyses were performed using one-way ANOVA with Dunnett's multiple comparison test (parametric variables). Scale bar = 1 mm.

To determine the turning point in pericardial adhesion, identified as an intensive increase in the adhesion score of our model, we injected a talc solution into the pericardial cavities of mice and evaluated tissue structures at both the gross and histological levels. Adhesions around the myocardium were observed (Figure 1A), and adhesion scores were remarkably increased (Figure 1B) on day 3 after talc injection. The highest adhesion scores, indicating complete adherence to the epicardium, were observed after day 4 (Figure 1B). These histological findings were consistent with the adhesion scores, suggesting that crucial changes occurred at the cellular level around day 4 after talc injection. Next, we excised the chest cavity for histological analysis to avoid inflicting pericardial injury during the thoracotomy (Figure 1C, right). H and E-stained specimens displayed excessive cell invasion and fibrosis in the pericardial cavity from day 4 (Figure 1C, arrowheads). Masson's trichrome staining results confirmed intense production of extracellular matrix proteins connecting the epicardium and pericardium, such as collagen, whose level increased significantly after day 4 (Figure 1C, arrowheads). To investigate cell-based histological features, we comprehensively compared the histological images at each time point. Visualization of Masson's trichrome- and H and E-stained specimens at high-magnification displayed neutrophils in the early stages (day 2), extravasation of red blood cells into the pericardial space (day 3), and invasion of fibroblastic cells into the myocardial tissue (day 5) (Figures 1C,D). The pathological findings in model mice regarding collagen depositions onto the surface of heart tissues were similar to those in human specimens with adhesions in the heart (Figure 1E). The ratios of epicardial erosion defined as discontinuous epicardium significantly increased (*p* < 0.001) at the third hour after injection (Figure 1F), while intact epicardia stained by immunohistochemical staining with GPM6A, which is known as a marker of mesothelial cells (22), was observed in the pre-injected or blood-injected mice using a previously established bleeding model (19) (Supplementary Figure 1). Hemorrhage was confirmed using immunohistochemical staining with anti-fibrinogen beta chain (FGβ) antibodies. Expectedly, the FGβ was located in the pericardial cavity on day 1 (Figure 1G). These data indicated that our model reproduced the pathologies of epicardial injury in the early stage, including mesothelial erosion, bleeding, and inflammatory cell accumulation previously reported in humans and other animal models.

### Providing Myofibroblasts and Endothelial Cells From the Beating Heart

**Figure 2:**
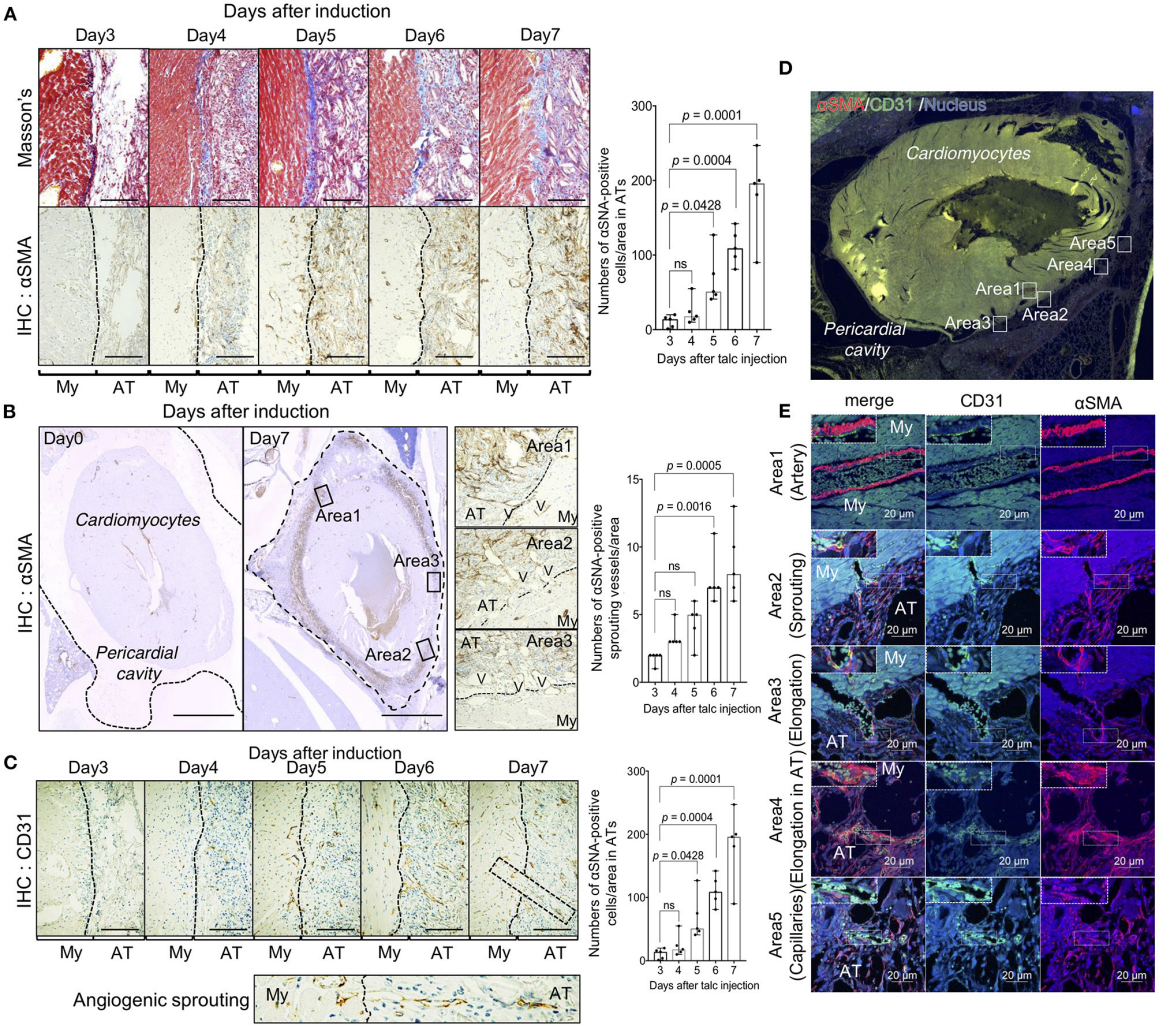
Unique angiogenic process from the heart during pericardial adhesion. **(A)** The left panels show the results of Masson's trichrome staining and α-SMA immunohistochemistry (IHC). The broken lines indicate the borders between myocardial (My) and adhesion (AT) tissues. Bar = 50 μm. The right panel shows quantified αSMA-positive cells in adhesion tissues (*n* = 5). Data are presented as means ± SE. Statistical analyses were performed using one-way ANOVA with Dunnett's multiple comparison test (parametric variables). **(B)** Left panels show the whole heart view of αSMA IHC before and after talc injection. The broken lines on day 0 indicate the pericardium. The middle panels show magnified views of areas 1–3 in the left panels. The broken lines indicate the borders between myocardial (My) and adhesion (AT) tissues. V indicates the presence of a vascular-like structure. Bar = 1 mm. The right panel shows quantified cell numbers of αSMA-positive bridging structures between myocardial and adhesion tissues (*n* = 5). Data are presented as means ± SE. Statistical analyses were performed using Kruskal-Wallis test with Dunn's multiple comparison test (non-parametric variables). **(C)** Tissue localization of CD31-positive endothelial cells. The lower panel shows an enlarged view of the sprouting vessels from myocardial tissues to adhesion tissues surrounded by broken lines in the upper right image. Bar = 50 μm. The right panel shows quantification of CD31-positive sprouting vessels per area in the pericardial surface (*n* = 5). Data are presented as means ± SE. Statistical analyses were performed using Kruskal-Wallis test with Dunn's multiple comparison test (non-parametric variables). My, myocardium; AT, adhesion tissue. **(D)** Immunofluorescence of whole heart stained using anti-αSMA (*red*) and CD 31 (*green*) antibodies. Nuclei were stained with Hoechst stain (*blue*). Bar = 1 mm. **(E)** Magnified views of areas 1–5 in **(D)**, representing quiescent, sprouting, and elongating blood vessels. Upper left dotted boxes in **(E)** show an enlarged view in the areas surrounded by broken lines. Bar = 20 μm. My, myocardium; AT, adhesion tissue.

To clarify the cellular mechanism of fibrosis observed in the pericardial cavity after day 4, we investigated the cellular behavior of αSMA-positive myofibroblasts responsible for collagen production during adhesion formation. As expected, few myofibroblasts were observed in the pericardial cavity on day 4, and the cell population was significantly increased (*p* = 0.0001) on day 7 in proportion to collagen deposition (Figure 2A). Interestingly, myofibroblasts were also located around the pericardial area, and the cell population tended to increase, suggesting that adhesion-related myofibroblasts might originate from the pericardial tissues. Based on our hypothesis, we quantified myofibroblasts migrating from the myocardial tissues to the pericardial cavity. As shown in the whole heart view illustrated in Figure 2B, myofibroblasts were more densely located around heart tissues than on the pericardial side. Notably, the abundance of vascular-like bridging myofibroblasts, which form lumen structures between cardiomyocytes and adhesion tissues, was significantly increased (*p* = 0.0005) on day 7 (Figure 2B). Based on our findings that the vascular-like structures were composed of myofibroblasts, we also investigated the localization of CD31-positive endothelial cells. A large number of endothelial cells were observed in adhesion and myocardial tissues on day 7 (Figure 2C). Notably, the abundance of CD31-positive sprouting vessels was remarkably increased from day 4 (Figure 2C), suggesting that angiogenesis and fibrosis might occur concomitantly during adhesion formation (Figures 2A,C). To validate our hypothesis, we conducted double immunofluorescence experiments using anti-CD31 and -αSMA antibodies. As shown in areas 1 of Figures 2D,E, several arterioles were composed of CD31-positive endothelial cells and αSMA-positive myofibroblasts as the inner and outer layers, respectively. Histological analysis of heart tissues on day 7 also revealed initial stages of sprouting (area 2), elongating vessels from myocardial tissues to adhesion tissues (area 3), and elongating vessels in adhesion tissues (area 4), which maintained the association of endothelial cells with αSMA-positive cells, and lumen structures with red blood cells (Figures 2D,E). A few myofibroblast-uncovered capillaries were observed in adhesion tissues (area 5 of Figure 2E).

**Figure 3:**
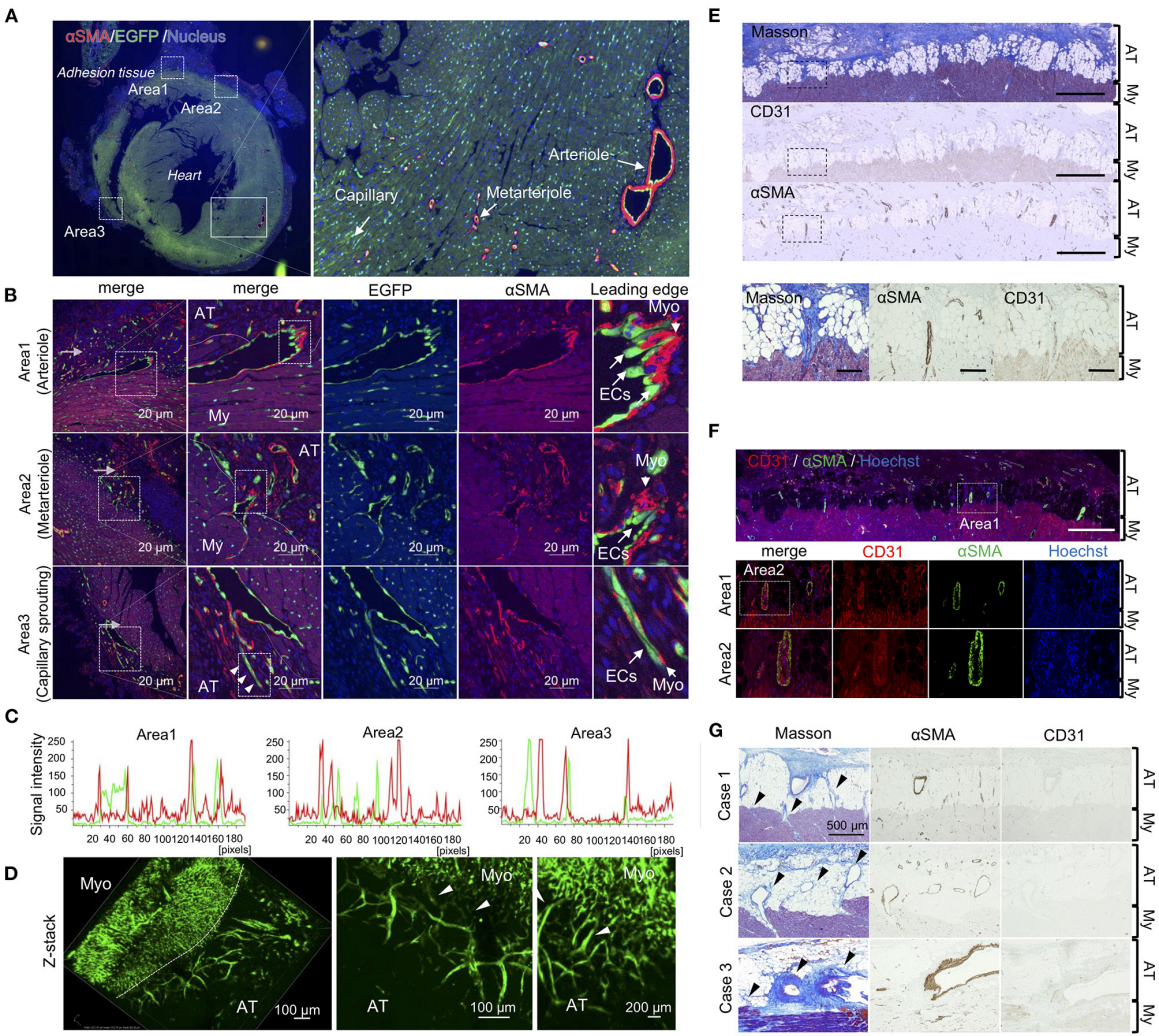
Unique angiogenic sprouting from heart in mice and humans. **(A)** Left panel shows immunofluorescence of whole heart stained using anti-αSMA (red) and EGFP (green) antibodies. Nuclei were stained with Hoechst stain (blue). The right panel shows a magnified view of the quiescent area surrounded by solid lines in the left panel. **(B)** Magnified views of areas 1–3 in the left of **(A)**. The rightmost images indicate enlarged views of areas surrounded by broken lines in the second panel from the left. Arrowheads indicate sprouting vessels from the arteriole. Bar = 20 μm. **(C)** Profiles of fluorescence signal intensities of αSMA (*red*) and EGFP (*green*) from areas 1–3 of the leftmost images in **(B)**. **(D)** Z-stacked images of heart tissue with adhesions in endothelial cell-specific EGFP-labeled mice. Heart tissue was excised and subsequently embedded into the type-I collagen gel, followed by obtaining z-stack three-dimensional images using A1 confocal microscopy. Arrowheads indicate vascular connective points between the heart and adhesions. My, myocardium; AT, adhesion tissue. **(E)** The upper panels show Masson's trichome staining and immunohistochemical (IHC) results for αSMA and CD31 in the human heart (Scale bar = 1 mm). The lower panels show magnified images of areas surrounded by broken lines in the upper panels (Scale bar = 200 μm). **(F)** Immunofluorescence co-staining and analysis of CD31 and αSMA in human pericardial adhesion tissues. The upper panel shows the immunofluorescence co-stained specimen illustrating identical areas corresponding to those observed in the Masson's trichrome-stained specimen image in **(A)** (Scale bar = 1 mm). The lower panels show high-magnification images of area 1 in the upper panel. Scale bar = 200 μm. **(G)** Histological evaluations (Masson's trichrome and IHC for CD31 and αSMA) of three patients with pericardial adhesion. Arrowheads indicate sprouting vessels from the heart. (Scale bar = 500 μm). My, myocardium; AT, adhesion tissue.

Next, we investigated whether arterioles and capillaries from the heart contributed to the development of fibrosis and vascular formation in the pericardial cavity. Using Rosa26^EGFP^; Cdh5^CreERT2^ mice that harbored EGFP-labeled endothelial cells, we traced endothelial cell behavior during adhesion. Arterioles and metarterioles were observed in the inner heart areas, and numerous capillaries were formed throughout heart tissues (Figure 3A). Immunofluorescence co-staining results also clearly indicated direct sprouting from arterioles and metarterioles (myofibroblasts located at the leading edge of sprouting vessels) (areas 1 and 2 in Figure 3B). Additionally, new capillary formation from arterioles was observed (area 3 of Figure 3B). However, no double-positive cells labeled with EGFP- and αSMA-derived fluorescence were present in heart tissues (Figure 3C), suggesting that endothelial cells could not be considered the origin of the myofibroblasts responsible for the development of fibrosis within 7 days of adhesion induction. In addition, three-dimensional tissue imaging based on confocal microscopy with z-stacking demonstrated that several EGFP-positive neoformed vasculatures sprouted in adhesion tissue from the vascular network in heart tissues (Figure 3D). To validate our findings obtained *via* the murine model, we evaluated the pathology of an autopsied patient who underwent cardiac surgery 6 months prior to the autopsy. Masson's trichrome staining results revealed the robust formation of adhesion tissues (indicated in blue) around layers of cardiomyocytes, while adipose tissues were partially observed within myocardial and adhesion tissues (Figure 3E). Interestingly, sprouting vessels from heart tissues were observed, composed of CD31-positive endothelial cells in the inner regions and αSMA-positive smooth muscle cells in the outer regions (Figure 3E). Tissue localization of CD31- and αSMA-positive cells during sprouting was confirmed by double immunofluorescence staining (Figure 3F). These angiogenic sprouting from the heart to adhesions were observed in all cases who underwent cardiac reoperation (Figure 3G). Taken together, the results indicated that endothelial cells and myofibroblasts originating from myocardial tissues might contribute to the development of post-surgical cardiac adhesions in humans.

### Gene Expression Profiling of Pericardial Adhesions in Mice

**Figure 4:**
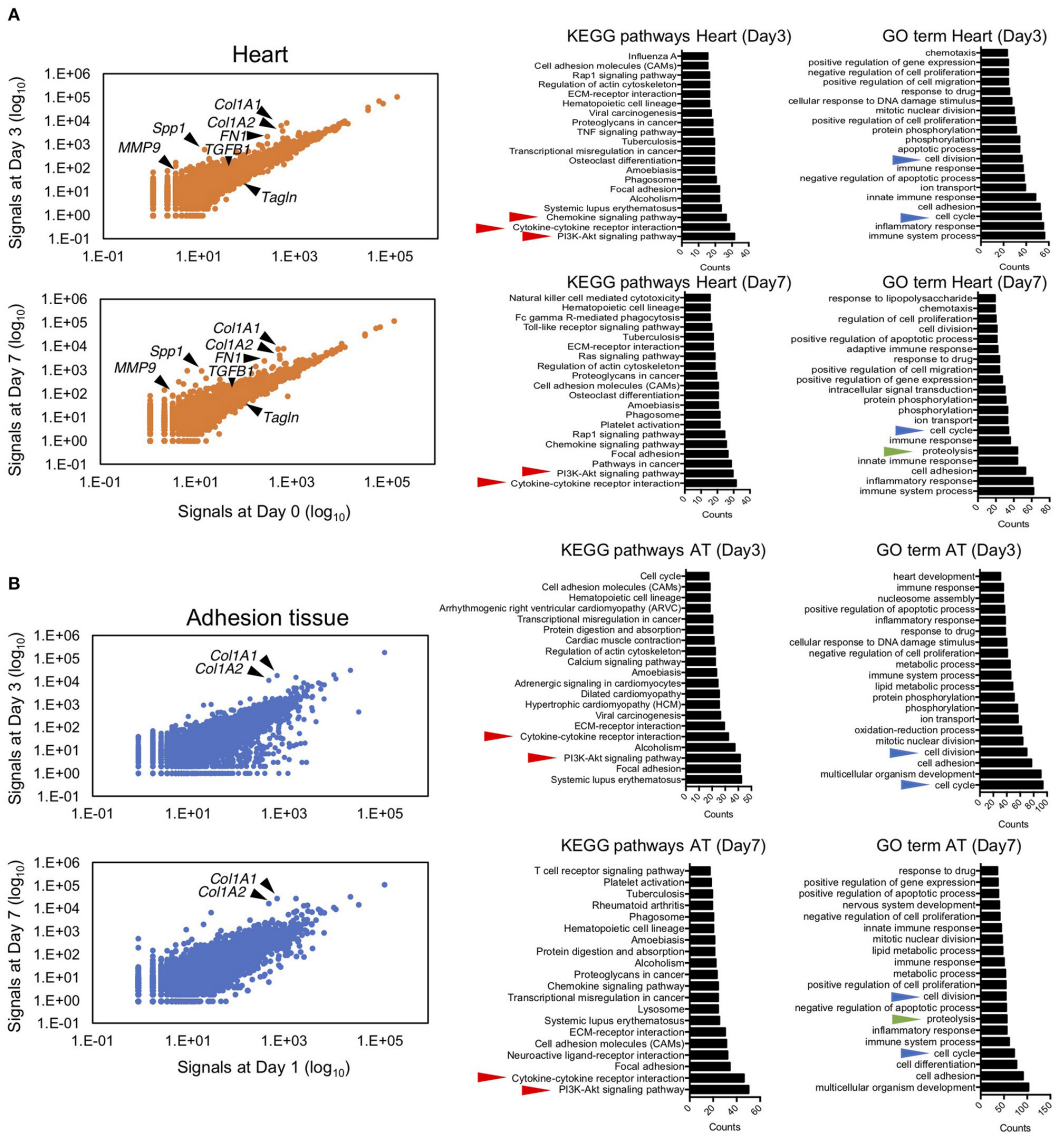
Next-generation sequencing of RNA from myocardial and adhesion tissues. **(A)** Scatter plot illustrating relative expression values obtained using RNA sequencing of myocardial tissues on days 3 and 7 after talc injection (left). The middle and right panels show the top 20 enriched gene ontologies based on KEGG and GO term database pathways, respectively. Red, blue, and green arrowheads indicate inflammation-, cell growth-, and proteolysis-related ontologies, respectively. **(B)** Scatter plot of relative expression values obtained by RNA sequencing of adhesion tissues on days 3 and 7 after induction (left). The middle and right panels show the top 20 enriched gene ontologies based on KEGG and GO term database pathways, respectively. Red, blue, and green arrowheads indicate inflammation-, cell growth-, and proteolysis-related ontologies, respectively.

To elucidate the molecular mechanism underlying pericardial adhesion, RNA sequencing was performed on samples obtained from heart and adhesion tissues. The scatter plot indicated that drastic changes did not occur in the expression levels of detected genes in heart tissues between days 3/0 and days 7/0 (Figure 4A). In heart tissues, 736 and 737 upregulated genes (>three-fold) and 231 and 220 downregulated genes (three-fold) were detected on days 3 and 7, respectively. To characterize the obtained gene sets, we determined Kyoto Encyclopedia of Gene and Genomes (KEGG) enrichment using the Database for Annotation, Visualization, and Integrated Discovery (DAVID) bioinformatics tool. KEGG pathway results revealed that inflammation-related genes played crucial roles in pathways for chemokine signaling, cytokine-cytokine receptor interaction, and PI3K-Akt signaling in both heart and adhesion tissues (Figure 4A). In contrast, 708 and 874 upregulated genes (>three-fold) and 220 and 788 downregulated genes (< three-fold) were found in adhesion tissues on days 3 and 7, respectively. Cytokine- and PI3k-Akt signaling-related genes were also enriched in both adhesion and heart tissues (Figure 4B), while the enriched gene patterns responsible for cytokine signaling differed considerably between heart and adhesion tissues (Supplementary Figure 2). Notably, Gene Ontology (GO) term analysis indicated genes associated with cell division-, cell cycle-, and proteolysis-related processes were enriched in both heart and adhesion tissues (Figures 4A,B), indicating that these genes might contribute to the formation of new blood vessels during adhesion. Downregulation of MYOCD signaling was seen as a common pathway between heart and adhesion tissues on day 3 using Ingenuity Pathway Analysis (IPA) (Supplementary Figure 3). IPA analysis also revealed upregulations of Leukocyte Extravasation and MMP9 signaling in the heart on day 3. Notably, upregulation of angiogenic signaling in the heart tissue on day 7 was also detected (Supplementary Figure 3).

### Cell Proliferation of Heart-Derived Myofibroblasts and Endothelial Cells

**Figure 5:**
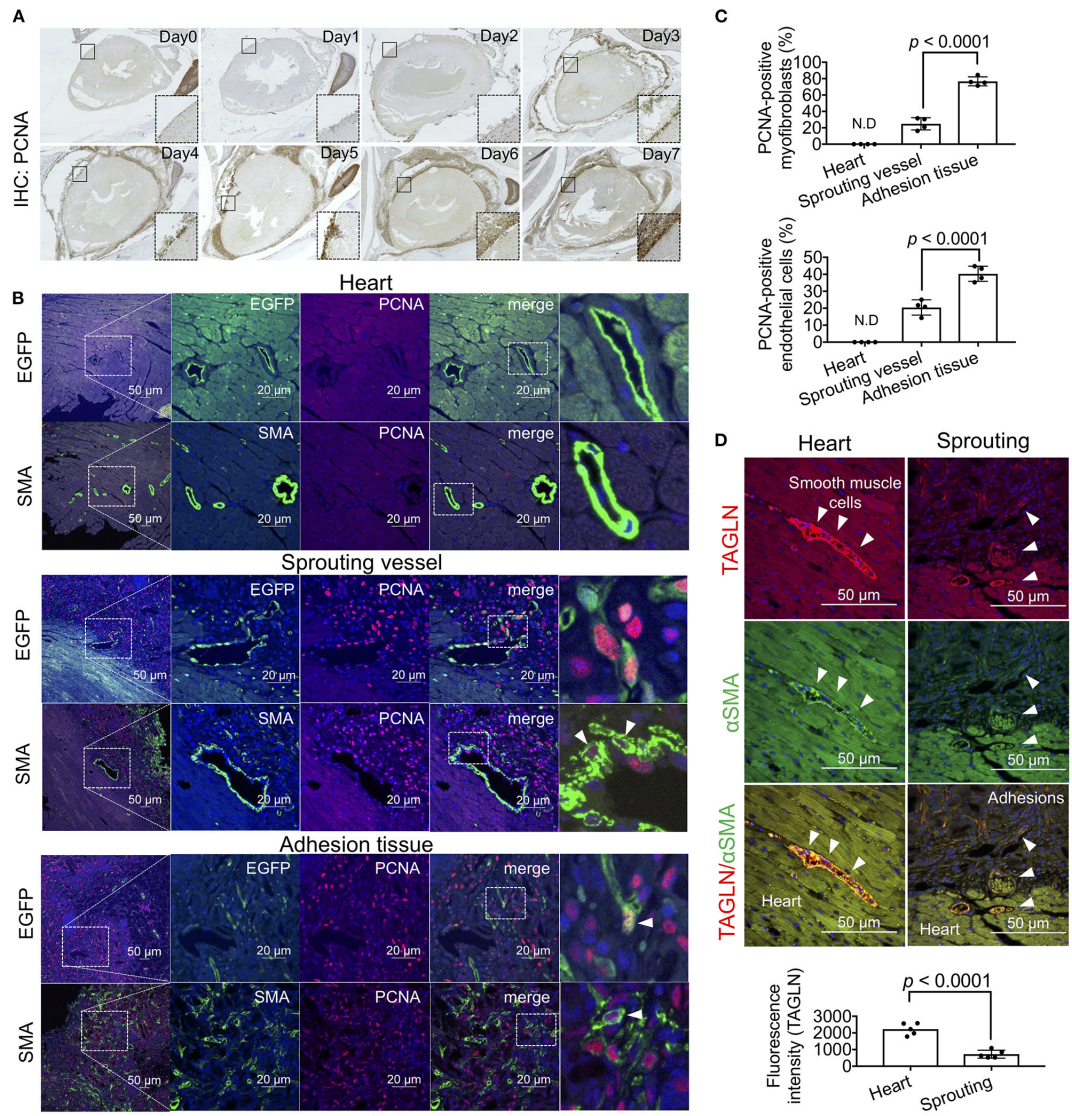
Cell growth of myofibroblasts and endothelial cells during adhesion formation. **(A)** The sagittal section of the chest cavity after immunohistochemistry (IHC) with anti-PCNA antibodies. The areas surrounded by broken lines indicate magnified images of the areas surrounded by solid lines. **(B)** Tissue localization of PCNA-positive endothelial cells and myofibroblasts within the myocardial tissues, sprouting vessels, and adhesion tissues. The rightmost panels include enlarged views of the areas surrounded by broken lines in the second panel from the right. Arrowheads indicate PCNA-positive cells. Scale bar = 20 μm. **(C)** Quantitative analysis of PCNA-positive myofibroblasts and endothelial cells in the heart, sprouting vessels, and adhesion tissues (*n* = 4). Data are presented as means ± SE. Statistical analyses were performed using a two-tailed *t*-test (parametric variables). **(D)** Immunohistochemical co-staining of αSMA and TAGLN of arterioles in heart tissue and sprouting vessels (upper). Arrowheads indicate TAGLN-positive smooth muscle cells. Quantification of TAGLN-derived fluorescence intensities of arterioles in the heart and sprouting vessels using NIS-Elements software. Data are presented as means ± SE. Statistical analyses were performed using a two-tailed *t*-test (parametric variables).

Based on the RNA sequencing results regarding cell cycle regulators, to visualize proliferative cells during adhesion formation, we performed immunohistochemical staining using anti-PCNA antibodies. A few PCNA-positive cells were observed around the injured surface of the heart on day 1, and the cell population gradually increased for 7 days (Figure 5A). From days 4–7, the abundance of proliferative cells was also markedly increased in adhesion tissues (Figure 5A). We next identified the types of PCNA-positive proliferative cells in heart and adhesion tissues using double immunofluorescence staining for endothelial cells and myofibroblasts. PCNA-derived fluorescence signals were not detected in EGFP-positive endothelial cells and αSMA-positive smooth muscle cells of heart tissues (Figure 5B, upper). However, PCNA was expressed in the nuclei of endothelial cells and myofibroblasts during vascular sprouting (Figure 5B, middle). Further, the populations of PCNA-positive proliferative endothelial cells and myofibroblasts were increased in adhesion tissues compared to those in sprouting vessels (Figure 5B, lower), with significantly different ratios of PCNA-positive endothelial cells (*p* < 0.0001) and myofibroblasts (*p* < 0.0001) between sprouting vessels and adhesion tissues (Figure 5C). Cell proliferation of both cells in sprouting vessels was confirmed by the immunofluorescence staining for ki67 (Supplementary Figure 4), suggesting that dedifferentiation of VSMCs from the contractile to the synthetic phenotype might have been induced. To test our hypothesis, immunohistochemical co-staining of TAGLN, a marker of smooth muscle cells, and αSMA was performed. We also found that while cardiac arterioles and sprouting vessels surrounded by TAGLN-stained smooth muscle cells were also observed, the fluorescence intensities of TAGLN were significantly decreased in sprouting vessels (*p* < 0.0001), rather than in cardiac arterioles. Our data indicate that proliferative dedifferentiated smooth muscle cells in arterioles of the heart are one of the origins of fibrosis in the pericardial cavity.

### Proteolysis-Dependent Tissue Remodeling in the Pre-angiogenic Stage

**Figure 6:**
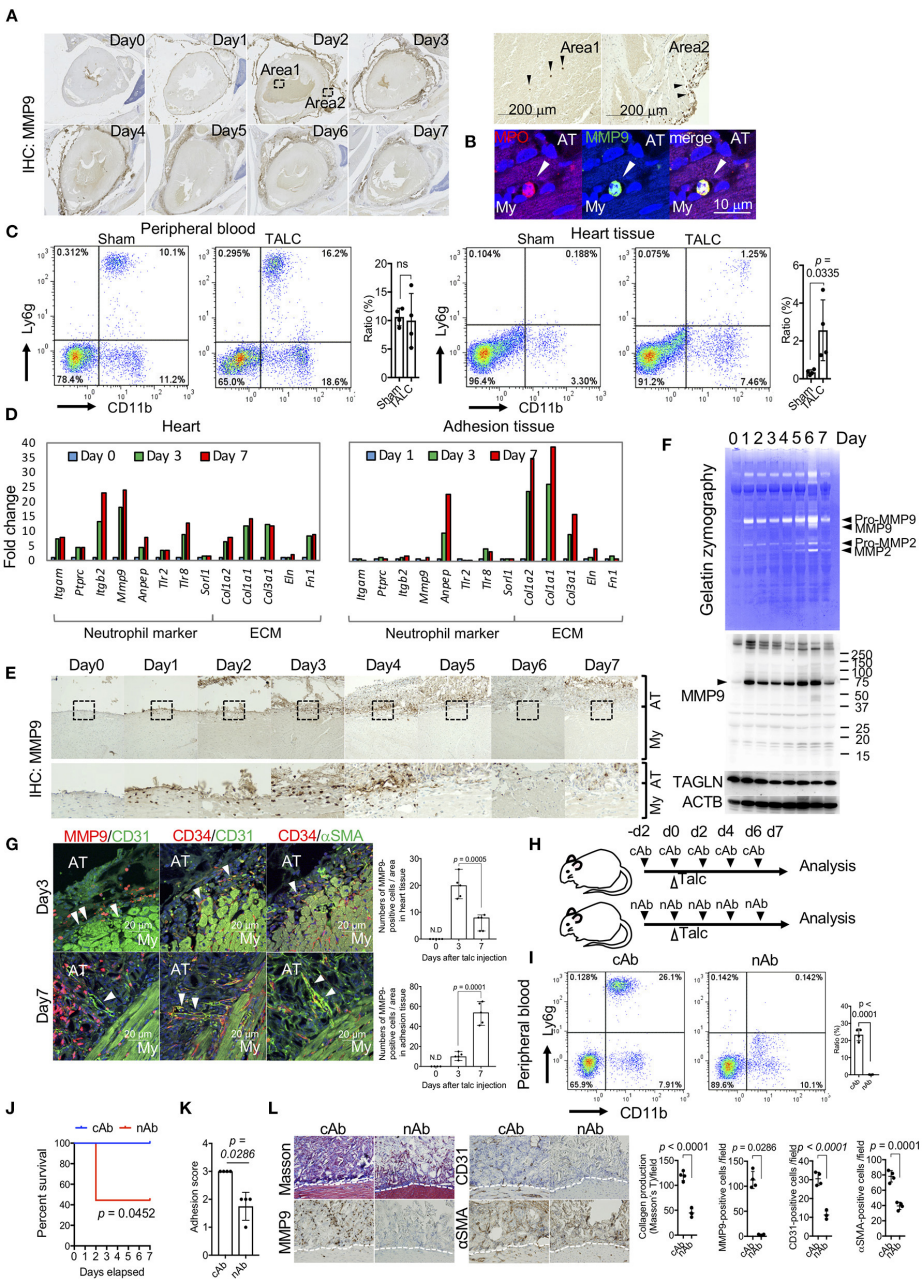
Matrix-metalloprotease-9 (MMP-9)-positive neutrophils on the surface of myocardial tissues is required for unique angiogenesis. **(A)** Whole heart view after immunohistochemistry (IHC) using anti-MMP9 antibodies. The right panels include magnified images (areas 1 and 2) of areas surrounded by broken lines in the left panels. Arrowheads indicate MMP9-positive cells in area 1 (surface of pericardial tissues) and area 2 (blood). **(B)** Immunofluorescence co-staining of MPO and MMP9 in the heart tissues of talc-injected mice on day 3. Arrowheads indicate MPO-positive cells. Scale bar = 10 μm. My, myocardium; AT, adhesion tissue. **(C)** Flow cytometric analyses of Ly6g^+^CD11b^+^ cell populations in the peripheral blood and heart tissue from sham or talc-injected mice (day 3). Right panel shows quantification of the Ly6g^+^CD11b^+^ population (%) in the sham and TALC groups. Data are presented as means ± SE. Statistical analyses were performed using a two-tailed *t*-test (parametric variables). **(D)** Left panel shows fold changes in mRNA expression of neutrophil markers and extracellular matrix of the heart (left) and adhesion (right) tissues on days 0 (blue), 3 (green), and 7 (red), calculated based on expression levels on day 0. **(E)** Temporal images of pericardial tissue surfaces stained with MMP9 antibody. The lower panels show magnified images of the areas surrounded by broken lines in the upper panels. My, myocardium; AT, adhesion tissue. **(F)** Gelatin zymography (*upper*) and western blot analyses for MMP9, TAGLN, and ACTB (*lower*) of tissue lysates from talc-injected mice within 7 days. **(G)** Immunofluorescence co-staining and analysis of MMP9, CD31, and αSMA in pericardial tissues on days 3 and 7. Arrowheads indicate blood vessels. Scale bar = 20 μm. Quantification of MMP9-positive cells in the heart and adhesion tissues on days 3 and 7 (n = 5). Data are presented as means ± SE. Statistical analyses were performed using a two-tailed *t*-test (parametric variables). **(H)** The administration schedule of anti-Ly6g neutralizing antibodies (nAb) or Isotype control antibodies (cAb). **(I)** Flow cytometric analyses of Ly6g^+^CD11b^+^ cell populations in the peripheral blood from the talc-injected mice with cAb or nAb treatments (day 3). Bar graph shows quantitative data of Ly6g^+^CD11b^+^ cells in cAb and nAb groups. Data are presented as means ± SE. Statistical analyses were performed using a two-tailed *t*-test (parametric variables). **(J)** Kaplan-Meier curves of cAb- (blue, *n* = 5) and nAb-treated (red, *n* = 9) mice within seven days after talc injections. Statistical analyses were performed using the Gehan-Breslow-Wilcoxon test. **(K)** Adhesion scores in the cAb- and nAb-treated mice on day 7 (*n* = 4). Data are presented as means ± SE. Mann-Whitney *U* (non-parametric variables). **(L)** Representative Masson's trichome (left), immunohistochemical staining for αSMA, CD31, and MMP9 (middle), and quantitative data of histology (right) in the cAb- and nAb-treated mice on day 7. My, myocardium; AT, adhesion tissue. Data are presented as means ± SE.; Statistical analyses were performed using a two-tailed *t*-test (parametric variables) for collagen production, IHC for CD31 and αSMA and Mann-Whitney *U* (nonparametric variables) for IHC for MMP9.

GO term and IPA analyses based on the RNA-seq data revealed enrichment of proteolysis-related genes, including *MMP9* (Figure 4). Since MMP9 is a proangiogenic factor that induces angiogenesis by subjecting the extracellular matrix to proteolysis (23), we investigated the tissue distribution of MMP9. MMP9-positive cells were observed on the surface of myocardial tissues on day 1 (Figure 6A). Although few MMP9-positive cells existed in the blood (area 1 of Figure 6A), these cells were distinctly accumulated on the surface of myocardial tissues on day 2 (area 2 in Figure 6A). Immunofluorescence co-staining of MMP9 and MPO, a marker of neutrophils, and flow cytometry for CD11b and Ly6g double-positive cells confirmed that the MMP9-positive cells were neutrophils (Figures 6B,C). Indeed, the expression levels of activated neutrophil marker genes, such as *itgam, ptprc, itgb2, mmp9, anpep, tlr2, tlr8, and sorl1*, were markedly upregulated in heart tissues on days 3 and 7. Conversely, these genes were not upregulated in adhesion tissues, characterized by overexpression of proteins encoded by extracellular matrix-associated genes, including *col1*α*2, col1*α*1, and co3*α*1* (Figure 6D and Supplementary Figure 5). Temporal histological analyses revealed that the accumulation of MMP9-positive neutrophils on the surface of myocardial tissues gradually decreased after day 5 (Figure 6E). Gelatin zymography and western blotting of tissue lysates from the heart also confirmed that MMP9 protein with protease activities was enriched after day 1, although protein levels of TAGLN (a marker of VSMCs) decreased with the progression of adhesion formation (Figure 6F). Immunofluorescence analysis also revealed the presence of MMP9-positive neutrophils around CD31- and CD34-double-positive blood vessels at the injured surface of myocardial tissues on day 3. After the occurrence of bleeding and the initial stages of collagen production in the pericardial cavity, the neutrophils gradually migrated into the adhesion tissues. The angiogenic sprouting of CD34/CD31 double-positive vessels covered by myofibroblasts occurred on the adhesion side (Figure 6G). To investigate the roles of MMP9-positive neutrophils, talc was administered to mice treated with isotype control antibodies- (cAb) or Ly6g neutralizing antibodies (nAb) (Figure 6H). Depletion of neutrophils was confirmed by flow cytometry for CD11b and Ly6g (Figure 6I). Fifty-five percentage of neutrophil-depleted mice could not survive talc injection, although control mice were not affected (Figure 6J). Further investigation of the surviving nAb-treated mice revealed that adhesion score, collagen deposition, and the number of angiogenic sprouting were significantly suppressed by depletion of neutrophils (Figures 6K,L). These findings indicate that MMP-9-positive neutrophils contribute to survival and adhesion formation through neovascularization by enabling remodeling of the collagen scaffold in myocardial tissues.

### MMP, PI3K, and SMAD Signaling-Dependent Angiogenic Sprouting Is Essential for Adhesion Formation

**Figure 7:**
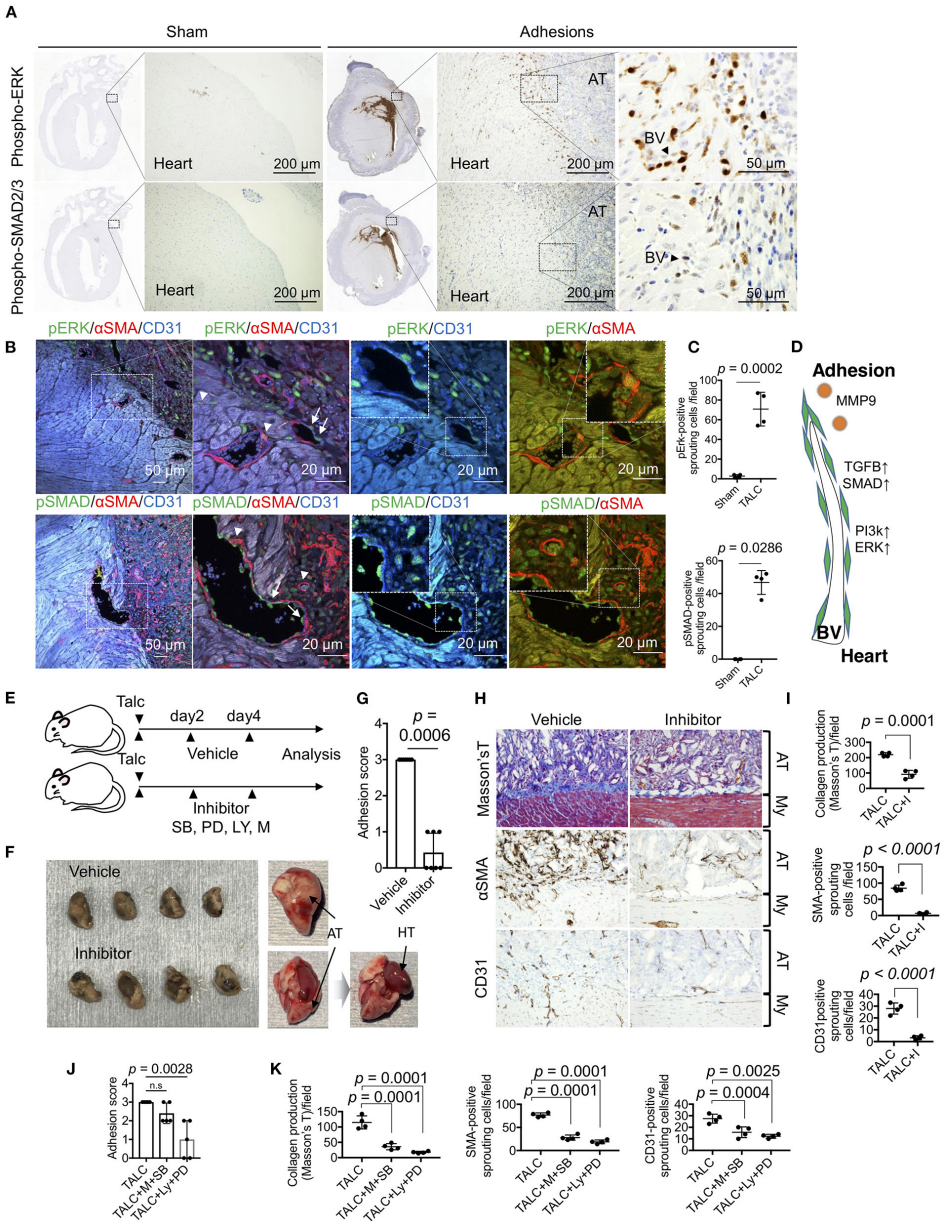
Sprouting vessel-specific cell signaling is required for adhesion formation. **(A)** Immunohistochemical (IHC) staining for phospho-ERK (pERK) and phosoho-SMAD2/3 (pSMAD) in the whole heart tissues from the sham group and talc injection group. (Scale bar = 200 μm). The areas surrounded by broken lines indicate magnified images. (Scale bar = 50 μm). AT, adhesion tissue; BV, blood vessel. **(B)** Tissue localization of pERK and pSMAD-positive endothelial cells and myofibroblasts within the sprouting vessels and adhesion tissues (Scale bar = 50 μm). The areas surrounded by broken lines indicate magnified images. (Scale bar = 20 μm) The arrowheads and arrows indicate pERK-positive myofibroblasts and pERK-positive endothelial cells within the sprouting vessels and adhesion tissues. **(C)** Quantitative analysis of pERK- and pSMAD-positive sprouting cells between myocardium and adhesion tissues (*n* = 4). Data are presented as means ± SE. Statistical analyses were performed using a two-tailed *t*-test (parametric variables) for IHC for pErk and Mann-Whitney *U* (non-parametric variables) for IHC for pSMAD. **(D)** Diagram depicting the molecular mechanism of angiogenic sprouting from the myocardium to adhesion tissues. BV, blood vessel. **(E)** The administration schedule of chemical inhibitors for pericardial adhesion mice. Chemical compounds (mixtures of SB: SB431542; PD: PD98059; LY: LY294002; M: Marimastat) were administered every 2 days for 6 days. **(F)** Excised hearts of vehicle- and inhibitor-treated group. Right panels show unfixed excised heart tissue. Inhibitor-treated heart tissues were easily divided from the pericardium. Arrows indicate adhesion tissues and heart tissues. AT, adhesion tissue; HT, heart tissue. **(G)** Adhesion scores of the excised heart tissues from vehicle- and inhibitor-treated groups (*n* = 7). Data are presented as means ± SE. Statistical analyses were performed using Mann-Whitney *U* (non-parametric variables) **(H)** Masson's trichome and immunohistochemical staining for αSMA and CD31 of vehicle- and inhibitor-treated groups (Bar = 50 μm). My, myocardium; AT, adhesion tissue. **(I)** The quantifications of collagen productions stained by blue, αSMA- and CD31-positive sprouting cells in the pericardial cavity from vehicle- and inhibitor-treated groups (*n* = 4). Statistical analyses were performed using unpaired Student's *t*-test. **(J)** Adhesion scores of the excised heart tissues from vehicle-, combination of M+SB-, and LY+PD-treated groups (*n* = 5). Data are presented as means ± SE. Statistical analyses were performed using Kruskal-Wallis test with Dunn's multiple comparison test (non-parametric variables). **(K)** The quantifications of collagen productions stained by blue, αSMA- and CD31-positive sprouting cells in the pericardial cavity from vehicle-, combination of M+SB-, and LY+PD-treated groups (*n* = 5). Data are presented as means ± SE. Statistical analyses were performed using one-way ANOVA with Dunnett's multiple comparison test (parametric variables).

To identify the critical cell signaling responsible for angiogenic sprouting, we focused on the PI3k/ERK and TGF-β/SMAD signaling, respectively known as master regulators of cell growth and fibrosis. Phosphorylated ERK- and SMAD2/3-positive cells were observed in the heart tissues of talc-treated mice, but not sham groups (Figure 7A). The fluorescence signals derived from phospho-ERK and SMADs were specifically detected in the CD31 or αSMA-positive sprouting vessels (Figure 7B). Compared to sham groups, the numbers of phospho-ERK and SMAD-positive sprouting vessels were significantly increased in talc-treated mice compared to sham groups (Figure 7C). To elucidate whether ERK or SMAD signaling is critical for angiogenic sprouting, talc-injected mice were treated with MEK/ERK inhibitor (PD98059) or TGF-β/SMAD inhibitor (SB431542). However, there was no difference between inhibitor and vehicle groups (Supplementary Figure 6). Our RNA-seq data also showed activation of PI3K/Akt, a master regulator of positive regulation of cell cycle and cell survival, in cardiac tissues (Figure 4A). In addition, MMP9, a powerful activator of TGF-β/SMAD signaling, was produced during angiogenic sprouting (Figure 6). Based on these data, we hypothesized that not only PI3k/ERK and TGF-β/SMAD signals but also MMP and PIk3/Akt signals might be essential for angiogenic sprouting during adhesions (Figure 7D). To address our idea, we treated the adhesion-induced mice with inhibitor mixtures (PD98059, SB431542, LY294002, and Marimastat) every 2 days (Figure 7E). As shown in Figure 7F, inhibitor mixture-treated mice exhibited a noticeable reduction of adhesion formation on the surface of heart tissues in the gross levels, although a dramatic change was not seen in groups of mice treated with a single inhibitor (Supplementary Figure 6). Adhesion score also significantly decreased when treated with the inhibitor mixture (*p* = 0.0006) (Figure 7G). Additionally, collagen productions on the heart surface and the number of αSMA and CD31-positive sprouting vessels were significantly decreased by the inhibitor mixture treatment (Figures 7H,I). Finally, to clarify the importance of PI3k/ERK and TGF-β/SMAD/MMP axes, adhesion-induced mice were treated with two-inhibitor cocktails (LY294002+ PD98059 and Marimastat+SB431542) every 2 days. The mixture of LY294002 and PD98059 significantly decreased adhesion scores, while treatment with a mixture of Marimastat and SB431542 tended to decrease the adhesion score (Figure 7J). Histological investigations revealed that both cocktails inhibited collagen production and angiogenic sprouting (Figure 7K). These data indicate that MMP9/TGF-β/SMAD and PI3K/ERK signaling activations are critical for angiogenic sprouting, followed by pericardial adhesions.

## Discussion

**Figure 8:**
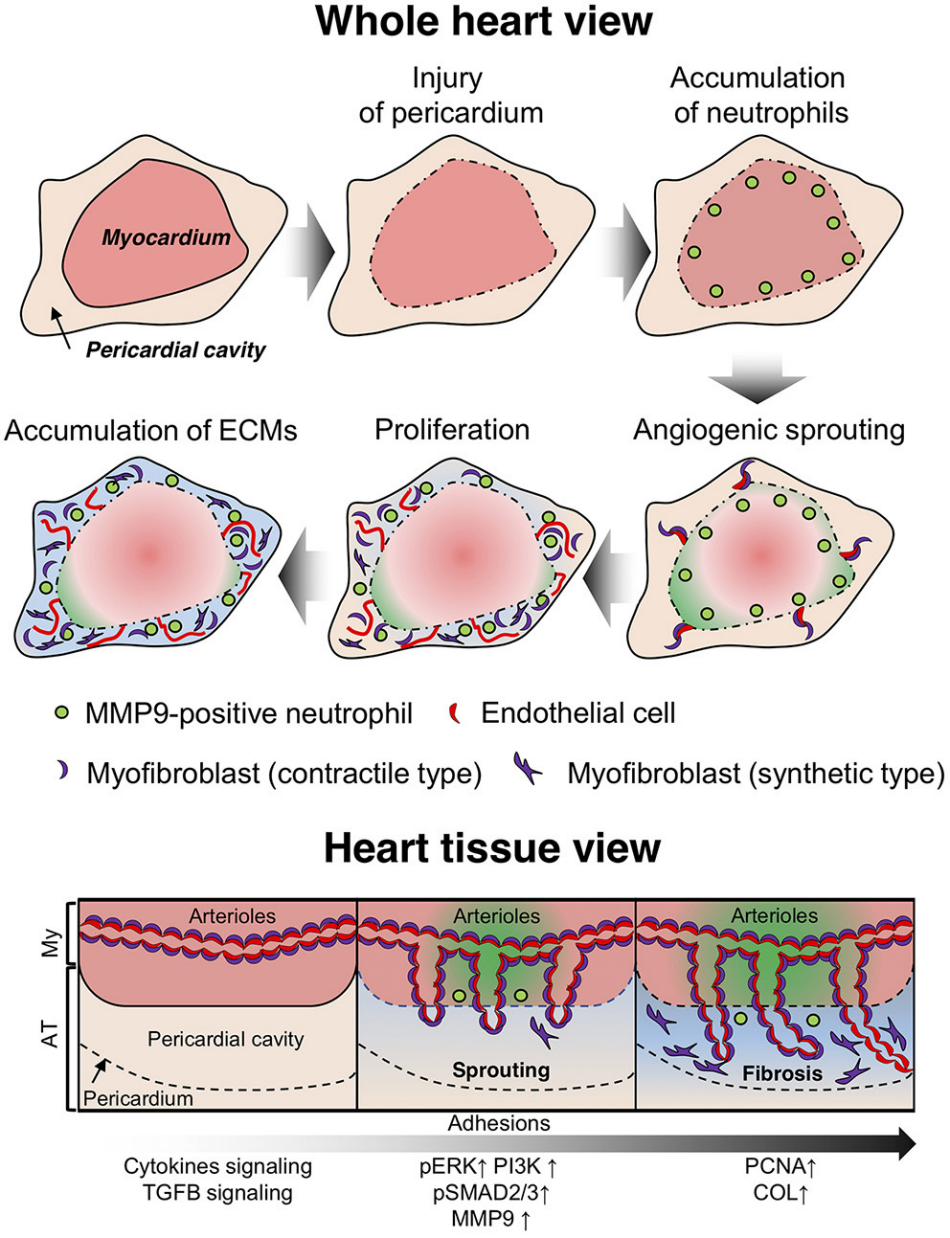
Diagram depicting the molecular mechanism of adhesion formation in the pericardial cavity. After infliction of epicardial injury, several types of immune cells (including neutrophils) accumulate on the surface of heart tissues to enable tissue remodeling via secretion of proteolytic enzymes. Arterioles and capillaries in the heart are excessively sprouted into the pericardial space. Then, smooth muscle cells with a contractile phenotype transition to a hyperproliferative and synthetic phenotype, which potentially contribute to fibrosis via production of extracellular matrix (ECM) proteins. The endothelial cells in the arterioles also exhibit a hyperproliferative phenotype and promote neovascularization in adhesion tissues (whole heart view). Arterial angiogenic sprouting occurs by maintaining vascular coverage with myofibroblasts (heart tissue view).

Several prevention methods have been developed for post-operative pericardial adhesions based on the observations in humans and animal models that bleeding, inflammation, and fibrosis are involved in the adhesion process (24). However, time-course histological assessments have not been conducted with quantification and molecular profiling during adhesion formation. Our murine model enabled the visualization of the adhesion process in the pericardial cavity (Figure 8) and helped elucidate its molecular mechanism using genetic modification technology. We observed the recruitment of neutrophils to the epicardial surface after the induction of pericardial injury, followed by remarkable vascular malformation with myofibroblast coverage from myocardial tissues during adhesion formation, contributing to fibrosis and blood supply into the pericardial space via the new blood vessels generated therein. The adhesions, separated by a fibrous layer from myocardium tissues, remained for at least 6 months in the pericardial regions (Supplementary Figure 7). The adhesions shared similar features with those of the autopsied patient in terms of the existence of rich blood vessels, the disappearance of myofibroblasts in the adhesion tissues, and adipose tissues formation around myocardial tissues (Figures 3E-G). Our murine model exhibited signs of multiple inflammatory reactions, such as epicardial injury, bleeding, and fibrosis, similar to those reported in previous studies conducted with humans or other animal models (24, 25). Thus, we demonstrated that the murine one-shot injection model was a powerful tool for investigating the molecular mechanism underlying pericardial adhesion formation.

Angiogenic development has been well characterized using a retinal model of angiogenesis in mice (10–13). These studies have shown that VEGF-A, a prime inducer of angiogenesis, guides endothelial cell migration toward peripheral regions, followed by vascular remodeling, such as arterialization. During the maturation and vein remodeling process, VSMCs are intensively recruited while the abundance of proliferative endothelial cells is subsequently decreased (13). Nevertheless, angiogenic processes during pericardial adhesion significantly differ from those observed during the developmental stage. Proliferative endothelial cells observed during sprouting were constantly wrapped by αSMA-positive cells, and red blood cells were contained in the vessels (Figure 2). Additionally, myofibroblasts located at the forefront of sprouting vessels played a crucial role as the leading cells of angiogenic sprouting (Figure 3B). Evidence of the generation of new blood vessels with αSMA-positive cells coverage in myocardial tissues has been recently reported, and αSMA-positive arterioles were discovered in newly formed vascular networks (26). αSMA-positive arterioles were also observed in skeletal muscles and heart tissues of mice with adeno-associated virus-mediated VEGF overexpression (27). The authors reported that VEGF-induced formation of new arterioles in the heart occurred to a lesser extent than in skeletal muscle. However, whether myofibroblasts covered the newly formed blood vessels following angiogenesis or whether vessel formation occurred via association between endothelial cells and myofibroblasts remains to be determined. Based on time-course experiments, our histological data demonstrated the occurrence of αSMA-positive cells-guided vascular formation along with red blood cell contents (Figure 2). Tracing analyses using endothelial cell-specific EGFP-expressing mice also revealed that endothelial-mesenchymal transition rarely occurred within 7 days, and each proliferated cell migrated into adhesion tissues (Figure 3). These results suggest that maintaining the vascular lumen structures of the arterioles may be essential for pathological neovascularization to avoid severe hypoxia during tissue repair, and direct recruitment of αSMA-positive cells from arterioles in myocardial tissues may be efficient for immediate adhesion formation.

The transition of VSMCs from the contractile to the synthetic phenotype, which exhibits high proliferative/migrative activities and overexpression of extracellular matrix-associated proteins, has been well characterized in neointimal formation during atherosclerosis (28). Multiple studies using lineage-tracing and gene expression analyses have demonstrated that plaque cells originating from dedifferentiated VSMCs are characterized by downregulated expression of *MYOCD, ACTA2, Tpm1, Tagln, Cnn1, ltga7, Des*, and *MYH11*, and upregulated expression of *Vim, Myh9, CD44, MMP3*, and *collagen*, which contribute to the development of vascular fibrosis *via* promotion of conversion to the synthetic phenotype (28–32). Additionally, TGF-β signaling is an essential regulator of VSMC dedifferentiation (33). Both our histological and RNA sequencing analyses demonstrated the conversion of VSMCs in the arteriole to proliferative myofibroblasts (Figure 5), downregulated expression of contractile marker genes, and upregulated expression of synthetic marker genes, as well as *TGFB1* and *TGFB-induced (TGFBI)* expression (Supplementary Figures 8A,B) and SMAD2/3 activation (Figure 7) in heart tissues during adhesion formation. Western blotting also confirmed that activation of SMAD2 in murine endothelial cells was induced by exposure to lysates from adhesion tissue (Supplementary Figure 8C). Proliferative αSMA-positive mural cells stained with anti-PCNA and -Ki67 antibodies were observed in the sprouting vessels from heart tissues to adhesion. These results suggest that analogical signaling and pathology exist between atherosclerosis and pericardial adhesions, and TGF-β-induced acquisition of the VSMC synthetic phenotype may also be essential for fibrosis in the pericardial cavity.

Comprehensive gene expression analysis also revealed that multiple cytokine signaling pathway-related genes that exhibit potential activities for enabling transition into the synthetic type, such as *CCL, CXCL, IL*, and the *TNF* family, were markedly enriched in the adhesions. Additionally, MMPs, including MMP9 and MMP2, known as a potent inducer of angiogenesis (34, 35) and activator of TGF-β (36), were overexpressed in heart tissues at the pre-angiogenic stages (Figure 6 and Supplementary Figure 9). However, the expression of VEGF families remained unchanged in both myocardial and adhesion tissues within 7 days of induction (Supplementary Figure 10). We successfully identified ERK and SMAD signaling as the driving force for cardiac angiogenic sprouting (Figure 7). The rescue experiments using chemical inhibitors confirmed that MMP/TGF-β/SMAD and PI3K signaling are essential for unique angiogenic sprouting. Indeed, several studies have demonstrated that four chemical inhibitors effectively abrogate ectopic overgrowth and transition into the synthetic type of VSMCs (37–46). Additionally, we found that the accumulation of MMP9-positive neutrophils at early stages was required for mouse survival following angiogenesis and fibrosis, suggesting that MMP9-positive cells may play dual roles as tissue protectors against acute pericarditis as well as angiogenic inducers. Our data suggest that atherosclerosis-related proliferation and recruitment of VSMCs and endothelial cells derived from arterioles in heart tissue play crucial roles in the adhesion formation on the injured epicardium.

The current study has several limitations. Solid live-imaging data of the angiogenic process during adhesion formation using multiphoton excitation fluorescence microscopy will be needed to establish a complete story for angiogenesis from the heart to adhesion tissues. Further investigations focusing on signal transduction of sprouting vessels are warranted to identify critical factors that regulate adhesion-related unique angiogenesis and the role of neutrophils in mouse survival during adhesions. We will further demonstrate the importance of angiogenesis in pericardial adhesion using angiogenic activity-deficient mice or smooth muscle cell-deletion mice. Our novel findings regarding unique vascular formations from heart tissues will help develop therapeutic options for pericardial adhesions, as well as for treatments of ischemic heart diseases, such as myocardial regeneration, which require the provision of oxygen and nutrition via new blood vessels.

## Data Availability Statement

The datasets presented in this study can be found in online repositories. The names of the repository/repositories and accession number(s) can be found below: NCBI [accession: GSE182735].

## Ethics Statement

The studies involving human participants were reviewed and approved by Ehime University Internal Review Board (protocol no.2105012). Informed consent for participation was obtained based on the opt-out principle, as approved by the Ehime University Internal Review Board. The animal study was reviewed and approved by Ehime University Animal Care Committee (Project number: 05-RO-7-1 and 05-RO-42).

## Author Contributions

KN and TS wrote the manuscript. HI, KN, and TS designed the study. KK, KN, MK, TS, and YKo conducted the experiments and acquired the data. FS, HI, JM, KK, KN, MK, MO, TN, HK, NO, SH, TS, YKo, and YKu analyzed the data and revised the manuscript. All authors contributed to the article and approved the submitted version.

## Funding

This work was supported by Grants-in-Aid for Scientific Research (grant no.18K16396 to KN) from the Ministry of Education, Culture, Sports, Science and Technology, Japan.

## Conflict of Interest

The authors declare that the research was conducted in the absence of any commercial or financial relationships that could be construed as a potential conflict of interest.

## Publisher's Note

All claims expressed in this article are solely those of the authors and do not necessarily represent those of their affiliated organizations, or those of the publisher, the editors and the reviewers. Any product that may be evaluated in this article, or claim that may be made by its manufacturer, is not guaranteed or endorsed by the publisher.

## Abbreviations

VSMC: vascular smooth muscle cell
VEGF: vascular endothelial growth factor
TGFB: transforming growth factor B
EGFP: enhanced green fluorescent protein
αSMA: alpha smooth muscle actin
PCNA: proliferating cell nuclear antigen
MMP: matrix metalloproteinase
GFP: green fluorescent protein
FGB: fibrinogen beta chain
HE: hematoxylin eosin
PBS: phosphate-buffered saline
HRP: horseradish peroxidase
DAB: diaminobenzidine
DAVID, Database for Annotation: Visualization and Integrated Discovery
KEGG: Kyoto Encyclopedia of Gene and Genomes
GO: Gene Ontology
Itgam: integrin subunit alpha M
Ptprc: protein tyrosine phosphatase receptor type C
Itgb2: integrin subunit beta 2
Anpep: alanyl aminopeptidase
Tlr2: toll-like receptor 2
Tlr8: toll-like receptor 8
Sorl1: sortilin-related receptor 1
Col1a2: collagen type 1 alpha 2 chain
Col1a1: collagen type 1 alpha 2 chain
Col3a1: collagen type 3 alpha 1 chain
MYOCD: myocardin
ACTA2: actin alpha 2
Tpm1: tropomyosin alpha-1 chain
Tagln: transgelin
Cnn1: calponin 1
itga7: integrin subunit alpha 7
Des, desmin, MYH11: myosin heavy chain 11
Vim: vimentin
Myh9: myosin heavy chain 9
TGFBI: transforming growth factor-beta induced
CCL: chemokine (C-C motif) chemokine ligand
CXCL: chemokine (C-X-C motif) chemokine ligand
IL: interleukin
TNF: tumor necrosis factor
IHC: immunohistochemistry
ECM: extracellular matrix.

## References

[1] Luciani N Anselmi A De Geest R Martinelli L Perisano M Possati G. Extracorporeal circulation by peripheral cannulation before redo sternotomy: indications and results. J Thorac Cardiovasc Surg. (2008) 136:572–7. 10.1016/j.jtcvs.2008.02.071 18805254

[2] Roselli EE Pettersson GB Blackstone EH Brizzio ME Houghtaling PL Hauck R. Adverse events during reoperative cardiac surgery: frequency, characterization, and rescue. J Thorac Cardiovasc Surg. (2008) 135:316–23, 323.e1–6. 10.1016/j.jtcvs.2007.08.060 18242260

[3] Song R Zhang L. Cardiac ECM: Its epigenetic regulation and role in heart development and repair. Int J Mol Sci. (2020) 21:1–20. 10.3390/ijms21228610 33203135 PMC7698074

[4] Mutsaers SE. The mesothelial cell. Int J Biochem Cell Biol. (2004) 36:9–16. 10.1016/S1357-2725(03)00242-5 14592528

[5] Fischer A Koopmans T Ramesh P Christ S Strunz M Wannemacher J. Post-surgical adhesions are triggered by calcium-dependent membrane bridges between mesothelial surfaces. Nat Commun. (2020) 11:3068. 10.1038/s41467-020-16893-3 32555155 PMC7299976

[6] Nkere UU Whawell SA Sarraf CE Schofield JB Thompson JN Taylor KM. Perioperative histologic and ultrastructural changes in the pericardium and adhesions. Ann Thorac Surg. (1994) 58:437–44. 10.1016/0003-4975(94)92224-1 8067846

[7] Leak LV Ferrans VJ Cohen SR Eidbo EE Jones M. Animal model of acute pericarditis and its progression to pericardial fibrosis and adhesions: ultrastructural studies. Am J Anat. (1987) 180:373–90. 10.1002/aja.1001800408 3425565

[8] Ishihara T Ferrans VJ Jones M Boyce SW Kawanami O Roberts WC. Histologic and ultrastructural features of normal human parietal pericardium. Am J Cardiol. (1980) 46:744–53. 10.1016/0002-9149(80)90424-5 7435384

[9] Folkman J. Seminars in Medicine of the Beth Israel Hospital, Boston. Clinical applications of research on angiogenesis. N Engl J Med. (1995) 333:1757–63. 10.1056/NEJM199512283332608 7491141

[10] Stahl A Connor KM Sapieha P Chen J Dennison RJ Krah NM. The mouse retina as an angiogenesis model. Invest Ophthalmol Vis Sci. (2010) 51:2813–26. 10.1167/iovs.10-5176 20484600 PMC2891451

[11] Okabe K Kobayashi S Yamada T Kurihara T Tai-Nagara I Miyamoto T. Neurons limit angiogenesis by titrating VEGF in retina. Cell. (2014) 159:584–96. 10.1016/j.cell.2014.09.025 25417109

[12] Sakaue T Maekawa M Nakayama H Higashiyama S. Prospect of divergent roles for the CUL3 system in vascular endothelial cell function and angiogenesis. J Biochem. (2017) 162:237–45. 10.1093/jb/mvx051 28981750

[13] Ehling M Adams S Benedito R Adams RH. Notch controls retinal blood vessel maturation and quiescence. Development. (2013) 140:3051–61. 10.1242/dev.093351 23785053

[14] Holmdahl L Ivarsson ML. The role of cytokines, coagulation, and fibrinolysis in peritoneal tissue repair. Eur J Surg. (1999) 165:1012–9. 10.1080/110241599750007810 10595602

[15] Schnaper HW Hayashida T Hubchak SC Poncelet A-C. TGF-β signal transduction and mesangial cell fibrogenesis. Am J Physiol Physiol. (2003) 284:F243–52. 10.1152/ajprenal.00300.2002 12529270

[16] Shu DY Butcher E Saint-Geniez M. EMT and EndMT: emerging roles in age-related macular degeneration. Int J Mol Sci. (2020) 21:4271. 10.3390/ijms21124271 32560057 PMC7349630

[17] Pankuweit S Wädlich A Meyer E Portig I Hufnagel G Maisch B. Cytokine activation in pericardial fluids in different forms of pericarditis. Herz. (2000) 25:748–54. 10.1007/PL00001993 11200123

[18] Elmadhun NY Sabe AA Lassaletta AD Dalal RS Sellke FW. Effects of alcohol on postoperative adhesion formation in ischemic myocardium and pericardium. Ann Thorac Surg. (2017) 104:545–52. 10.1016/j.athoracsur.2016.11.075 28262301

[19] Kojima A Sakaue T Okazaki M Shikata F Kurata M Imai Y. A simple mouse model of pericardial adhesions. J Cardiothorac Surg. (2019) 14:124. 10.1186/s13019-019-0940-9 31253183 PMC6599257

[20] Saeki N Imai Y. Reprogramming of synovial macrophage metabolism by synovial fibroblasts under inflammatory conditions. Cell Commun Signal. (2020) 18:188. 10.1186/s12964-020-00678-8 33256735 PMC7708128

[21] Li P Lu M Shi J Hua L Gong Z Li Q. Dual roles of neutrophils in metastatic colonization are governed by the host NK cell status. Nat Commun. (2020) 11:4387. 10.1038/s41467-020-18125-0 32873795 PMC7463263

[22] Bochmann L Sarathchandra P Mori F Lara-Pezzi E Lazzaro D Rosenthal N. Revealing new mouse epicardial cell markers through transcriptomics. PLoS ONE. (2010) 5:e11429. 10.1371/journal.pone.0011429 20596535 PMC2893200

[23] Bergers G Brekken R McMahon G Vu TH Itoh T Tamaki K. Matrix metalloproteinase-9 triggers the angiogenic switch during carcinogenesis. Nat Cell Biol. (2000) 2:737–44. 10.1038/35036374 11025665 PMC2852586

[24] Cannata A Petrella D Russo CF Bruschi G Fratto P Gambacorta M. Postsurgical intrapericardial adhesions: mechanisms of formation and prevention. Ann Thorac Surg. (2013) 95:1818–26. 10.1016/j.athoracsur.2012.11.020 23566646

[25] Ramasamy V Mayosi BM Sturrock ED Ntsekhe M. Established and novel pathophysiological mechanisms of pericardial injury and constrictive pericarditis. World J Cardiol. (2018) 10:87–96. 10.4330/wjc.v10.i9.87 30344956 PMC6189073

[26] Melly L Cerino G Frobert A Cook S Giraud MN Carrel T. Myocardial infarction stabilization by cell-based expression of controlled vascular endothelial growth factor levels. J Cell Mol Med. (2018) 22:2580–91. 10.1111/jcmm.13511 29478261 PMC5908097

[27] Kocijan T Rehman M Colliva A Groppa E Leban M Vodret S. Genetic lineage tracing reveals poor angiogenic potential of cardiac endothelial cells. Cardiovasc Res. (2021) 117:256–70. 10.1093/cvr/cvaa012 31999325 PMC7797216

[28] Basatemur GL Jørgensen HF Clarke MCH Bennett MR Mallat Z. Vascular smooth muscle cells in atherosclerosis. Nat Rev Cardiol. (2019) 16:727–44. 10.1038/s41569-019-0227-9 31243391

[29] Bondareva O Sheikh BN. Vascular homeostasis and inflammation in health and disease-Lessons from single cell technologies. Int J Mol Sci. (2020) 21:4688. 10.3390/ijms21134688 32630148 PMC7369864

[30] Rzucidlo EM Martin KA Powell RJ. Regulation of vascular smooth muscle cell differentiation. J Vasc Surg. (2007) 45(Suppl A):A25–32. 10.1016/j.jvs.2007.03.001 17544021

[31] Wright DB Trian T Siddiqui S Pascoe CD Johnson JR Dekkers BGJ. Phenotype modulation of airway smooth muscle in asthma. Pulm Pharmacol Ther. (2013) 26:42–9. 10.1016/j.pupt.2012.08.005 22939888

[32] Chaabane C Coen M Bochaton-Piallat M-L. Smooth muscle cell phenotypic switch: implications for foam cell formation. Curr Opin Lipidol. (2014) 25:374–9. 10.1097/MOL.0000000000000113 25110900

[33] Chen P-Y Qin L Li G Tellides G Simons M. Fibroblast growth factor (FGF) signaling regulates transforming growth factor beta (TGFβ)-dependent smooth muscle cell phenotype modulation. Sci Rep. (2016) 6:33407. 10.1038/srep33407 27634335 PMC5025753

[34] Nozawa H Chiu C Hanahan D. Infiltrating neutrophils mediate the initial angiogenic switch in a mouse model of multistage carcinogenesis. Proc Natl Acad Sci USA. (2006) 103:12493–8. 10.1073/pnas.0601807103 16891410 PMC1531646

[35] Shojaei F Singh M Thompson JD Ferrara N. Role of Bv8 in neutrophil-dependent angiogenesis in a transgenic model of cancer progression. Proc Natl Acad Sci USA. (2008) 105:2640–5. 10.1073/pnas.0712185105 18268320 PMC2268189

[36] Kobayashi T Kim HJ Liu X Sugiura H Kohyama T Fang Q. Matrix metalloproteinase-9 activates TGF-β and stimulates fibroblast contraction of collagen gels. Am J Physiol Lung Cell Mol Physiol. (2014) 306:L1006–15. 10.1152/ajplung.00015.2014 24705725 PMC4042193

[37] Hecht E Freise C Websky K V Nasser H Kretzschmar N Stawowy P. The matrix metalloproteinases 2 and 9 initiate uraemic vascular calcifications. Nephrol Dial Transplant. (2016) 31:789–97. 10.1093/ndt/gfv321 26333546

[38] Smiljanic K Obradovic M Jovanovic A Djordjevic J Dobutovic B Jevremovic D Marche P. Thrombin stimulates VSMC proliferation through an EGFR-dependent pathway: involvement of MMP-2. Mol Cell Biochem. (2014) 396:147–60. 10.1007/s11010-014-2151-y 25047892

[39] Wang C Wen J Zhou Y Li L Cui X Wang J. Apelin induces vascular smooth muscle cells migration *via* a PI3K/Akt/FoxO3a/MMP-2 pathway. Int J Biochem Cell Biol. (2015) 69:173–82. 10.1016/j.biocel.2015.10.015 26494002

[40] Ha YM Nam J-O Kang YJ. Pitavastatin regulates Ang II induced proliferation and migration *via* IGFBP-5 in VSMC. Korean J Physiol Pharmacol. (2015) 19:499–506. 10.4196/kjpp.2015.19.6.499 26557016 PMC4637352

[41] Dadlani H Ballinger ML Osman N Getachew R Little PJ. Smad and p38 MAP kinase-mediated signaling of proteoglycan synthesis in vascular smooth muscle. J Biol Chem. (2008) 283:7844–52. 10.1074/jbc.M703125200 18223258

[42] Numaga-Tomita T Shimauchi T Oda S Tanaka T Nishiyama K Nishimura A. TRPC6 regulates phenotypic switching of vascular smooth muscle cells through plasma membrane potential-dependent coupling with PTEN. FASEB J. (2019) 33:9785–96. 10.1096/fj.201802811R 31162976 PMC6704458

[43] Takahashi M Hayashi K Yoshida K Ohkawa Y Komurasaki T Kitabatake A. Epiregulin as a major autocrine/paracrine factor released from ERK- and p38MAPK-activated vascular smooth muscle cells. Circulation. (2003) 108:2524–9. 10.1161/01.CIR.0000096482.02567.8C 14581411

[44] Blanc A Pandey NR Srivastava AK. Synchronous activation of ERK 1/2, p38mapk and PKB/Akt signaling by H2O2 in vascular smooth muscle cells: potential involvement in vascular disease (review). Int J Mol Med. (2003) 11:229–34. 10.3892/ijmm.11.2.229 12525883

[45] Yu H-W Liu Q-F Liu G-N. Positive regulation of the Egr-1/osteopontin positive feedback loop in rat vascular smooth muscle cells by TGF-beta, ERK, JNK, and p38 MAPK signaling. Biochem Biophys Res Commun. (2010) 396:451–6. 10.1016/j.bbrc.2010.04.115 20417179

[46] Eguchi S Dempsey PJ Frank GD Motley ED Inagami T. Activation of MAPKs by angiotensin II in vascular smooth muscle cells. Metalloprotease-dependent EGF receptor activation is required for activation of ERK and p38 MAPK but not for JNK. J Biol Chem. (2001) 276:7957–62. 10.1074/jbc.M008570200 11116149

