# Supplementary material for: Unique Angiogenesis From Cardiac Arterioles During Pericardial Adhesion Formation
Source: Front Cardiovasc Med. 2022 Feb 3;8:761591. doi: 10.3389/fcvm.2021.761591 (PMC8852280; doi:10.3389/fcvm.2021.761591)
Supplement: Supplementary file 1 [file Data_Sheet_1.docx]

***Supplementary Material***

**Supplementary Figures and Tables**

**
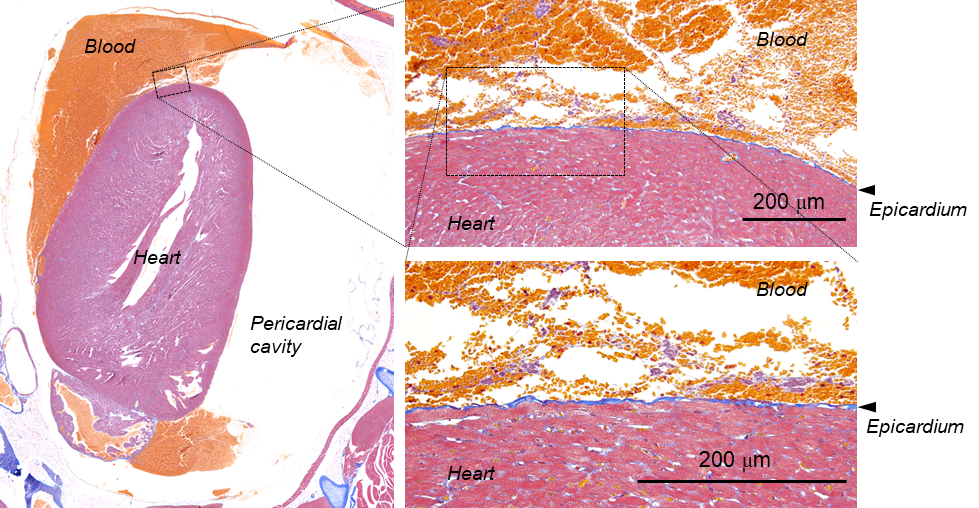
**

**Supplemental Figure 1. Histological analysis of the pericardial bleeding model in mice.**

Anesthesia and laparotomy were performed as per the methods described in the main text. Blood (300 µL) without anticoagulants collected from donor mice was injected into the pericardial cavity. The chest cavity was excised after the third hour and subjected to fixation with neutral buffered formalin. Masson’s trichrome staining was performed as per the methods described in the main text. Sagittal images of the chest cavity were acquired using the Keyence BZ-x800 microscope (*left panel*). The area surrounded by broken lines in the left panel was enlarged (*right upper panel*: x200 and *right lower panel*: x400). The arrowheads indicate the epicardium. Scale bar = 200 μm.

**Supplemental Figure 2. Maps illustrating the cytokine signaling pathways in heart or adhesion tissues on day 7 after talc injection.** Upregulated (>3 fold) and downregulated (<3-fold) expression of genes based on Mi-Seq RNA analysis was used for cytokine signaling analysis using the DAVID bioinformatics tool. Gene names labeled with red stars indicate enriched genes in the heart (*upper panel*) and adhesion (*lower panel*) tissues, identified using the Kyoto Encyclopedia of Genes and Genome (KEGG) database.

Heart day 7 cytokine

Adhesion tissue day 7 cytokine


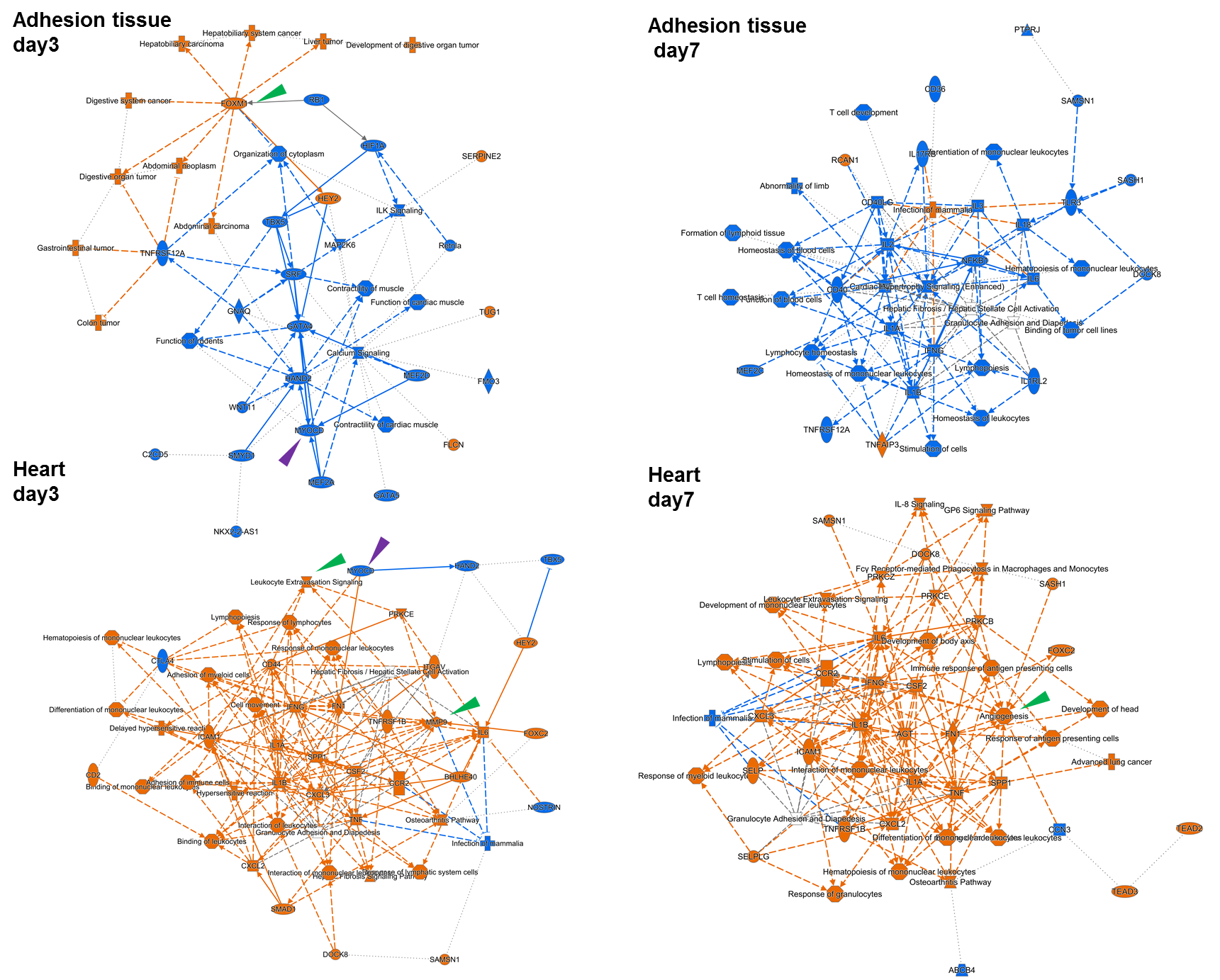


**Supplemental Figure 3. Graphical representation of signaling maps in the heart and adhesion tissue on days 3 and 7 after talc injection.** Upregulated and downregulated expression of genes (Cutoff range.; 0.3 up and -0.3 down for Log Ratio) based on Mi-Seq RNA analysis was used for IPA signaling analysis (IPA software version 01-19-02 (Qiagen Bioinformatics)). Signal names indicated with the purple arrowhead shows the common downregulated pathway. Signal names indicated with green arrowheads show the heart-specific activated pathways.


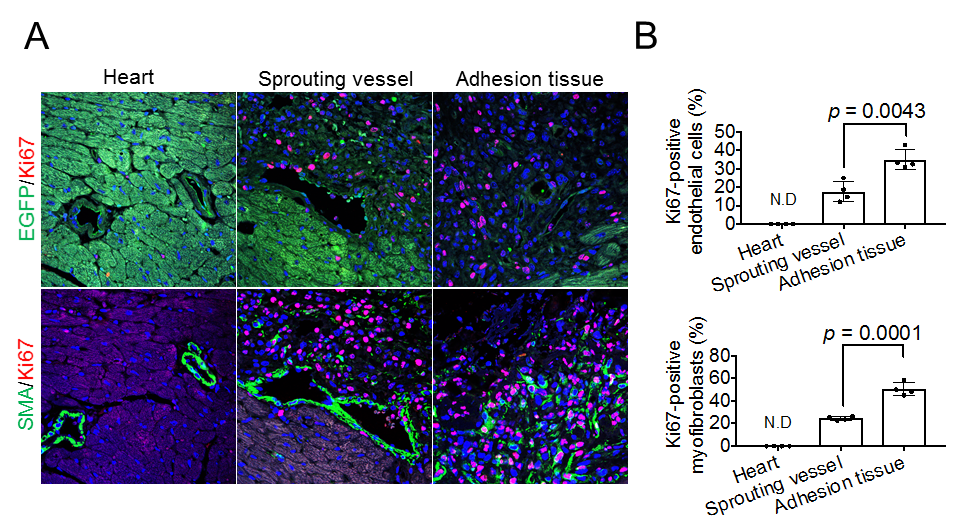


**Supplemental Figure 4. Evaluations of Ki67-positive proliferative endothelial cells and αsmooth muscle cells during adhesion formation.** A. Tissue localization of Ki67-positive endothelial and myofibroblastic cells in the heart, sprouting vessels, and adhesion tissue. Deparafinized talc-injected heart samples on day 7 were co-incubated with anti-Ki67 (Cell signaling., catalog no. 12202., 1:400 dilution) and anti-GFP (Nacalai Tesque., Kyoto., Japan. Catalog no. 04363-24., 1:400 dilution) or αSMA (abcam., catalog no. ab270251., 1:400 dilution) antibodies, overnight. After washing, Alexa Fluor 488-conjugated goat anti-mouse IgG (1:1,000)/Alexa Fluor 568-conjugated goat anti-rabbit IgG (1:1,000) were probed for two hours, followed by staining with Hoechst 33342. Fluorescence images were taken using an A1 confocal laser microscope (Nikon Co., Tokyo, Japan). B. Quantifications of Ki67-positive myofibroblasts and endothelial cells in the heart, sprouting vessels, and adhesion tissue (n = 4). Data are presented as means ± SE.; Two-tailed t-test (parametric variables).


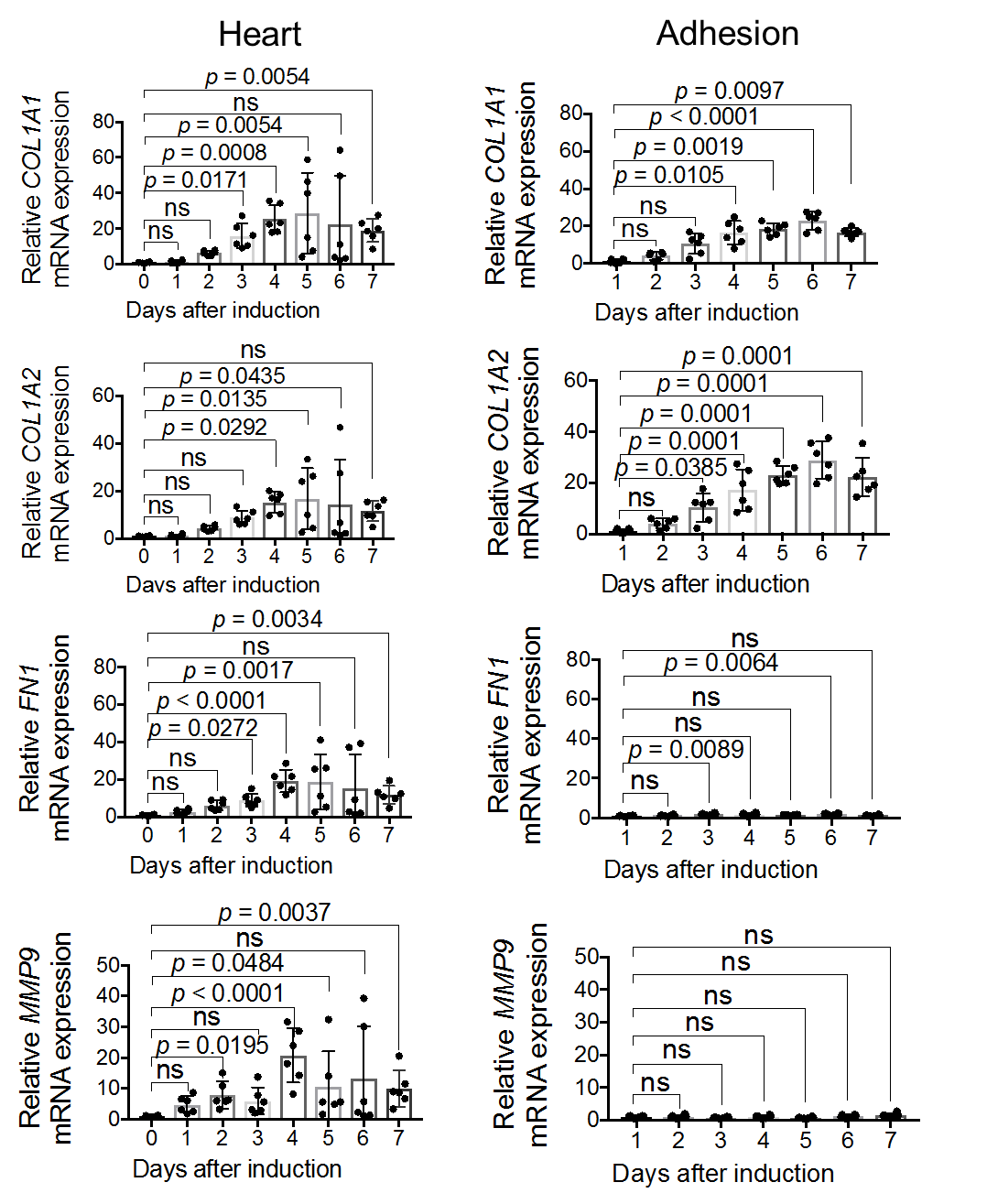
**Supplemental Figure 5. Quantitative analysis of tissue remodeling-related genes.** Extraction and purification of total RNA from adhesion or heart tissues were performed as per methods described in the main text. Reverse transcription-polymerase chain reaction (RT-PCR) was conducted using a high-capacity RNA-to-cDNA kit (Applied Biosystems, Foster City, CA, USA) with 1 μg total RNA, and first-strand synthesis was conducted according to the manufacturer’s instructions. Real-time PCR was performed using the 7500 Fast Real-Time PCR system (Applied Biosystems) with FastStart Universal SYBR Green Master (Rox) (Roche Diagnostics, Basel, Switzerland). The relative expression levels were calculated using the delta-delta Ct method. Expression levels of target genes were normalized to that of GAPDH. mRNA levels of *COL1A1, COL1A2, FN1*, and *MMP9* were quantified using the primers listed in Supplementary Table 1. Values are expressed as mean ± standard error (n = 6 in each group). Data are presented as means ± SE.; Kruskal–Wallis test with Dunn’s multiple comparison test (nonparametric variables).

**
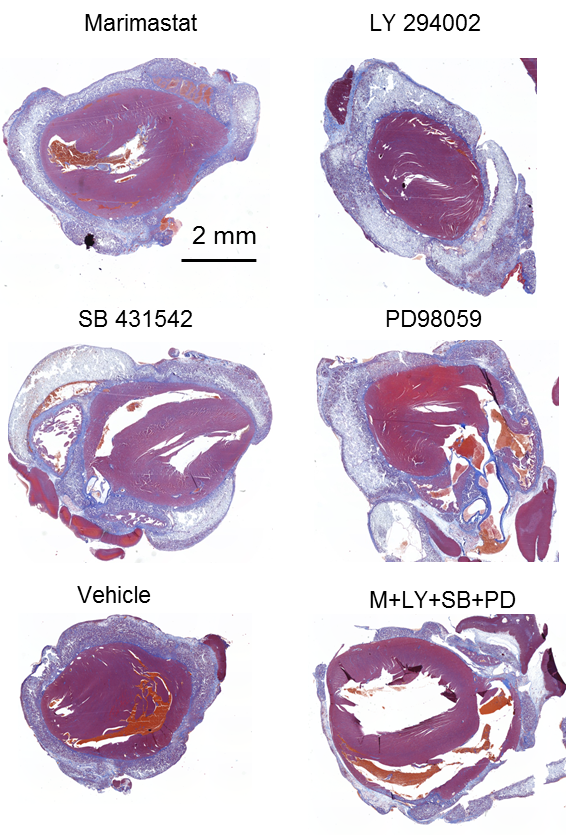
**

**Supplemental Figure 6. Masson’s trichrome staining of pericardial adhesions from chemical inhibitor-treated adhesion model mice.**

TGF-β type1 receptor inhibitor (385 μg/kg, SB431542), MEK/ERK pathway inhibitor (2.7 mg/kg, PD98059), PI3K-kinase inhibitor (1.2 mg/kg, LY294002), and MMPs inhibitor (25 mg/kg, Marimastat) were simultaneously or separately injected into the pericardial cavity. Mice were treated with inhibitors on day 0 and intraperitoneally administered on day 2 and day 4. All chemical inhibitors were dissolved in corn oil (total volume: 200 μL).

**
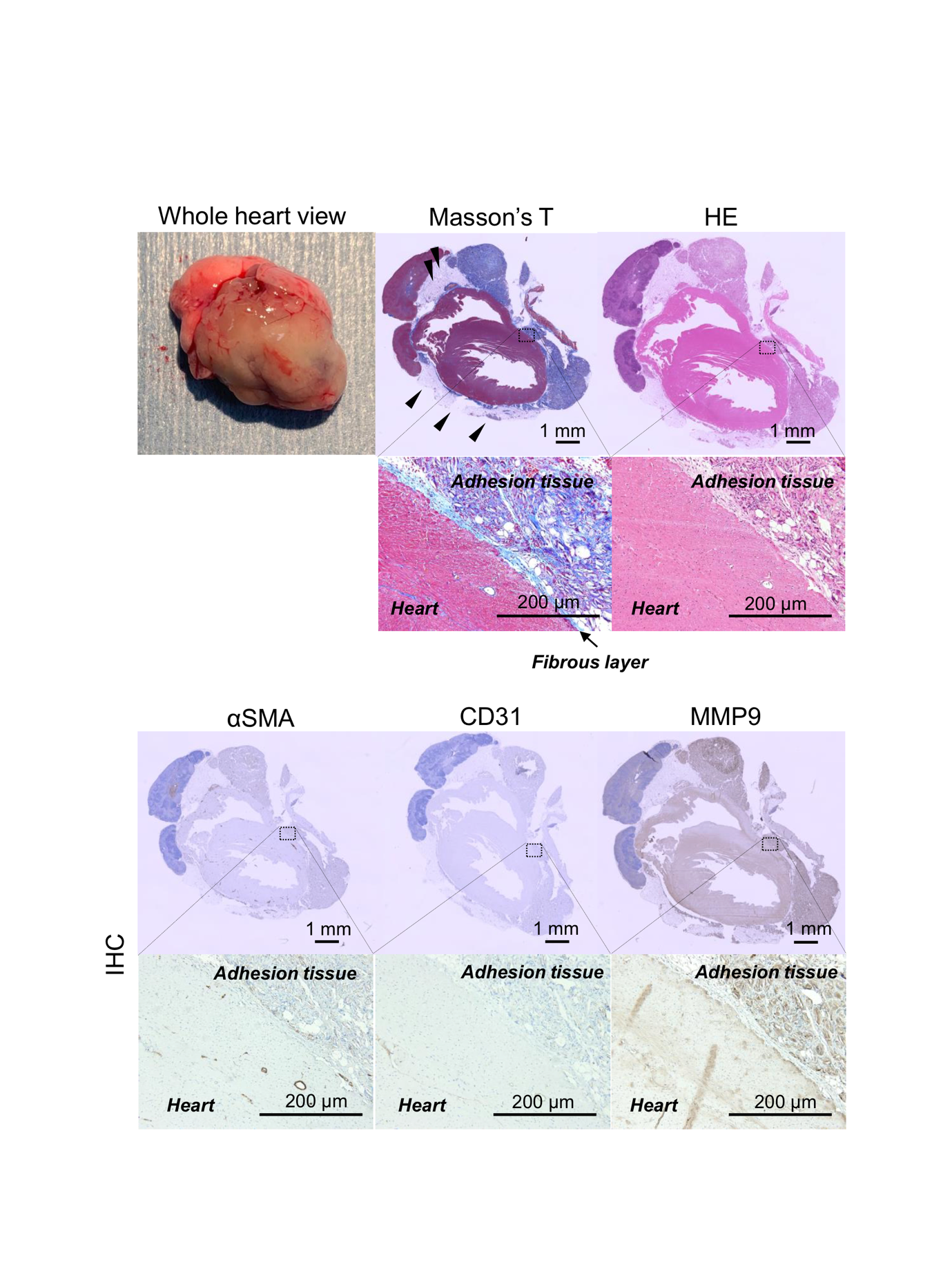
**

**Supplemental Figure 7. Histological evaluation of pericardial adhesions in mice six months after talc injection.** Whole heart view and Masson’s trichrome- and H&E-stained heart tissues (sagittal section of the chest cavity and enlarged views) in mice six months after talc injection (*upper panels*). The arrowheads indicate adipose tissue. The arrow indicates the fibrous layer that was newly formed at the border between heart and adhesion tissues. Localization of myofibroblasts, blood vessels, and MMP9-positive cells was assessed by immunohistochemical (IHC) staining with anti-αSMA (1:2000), anti-CD31 (1:200), and anti-MMP9 (1:1000) antibodies (*lower panels*).

**
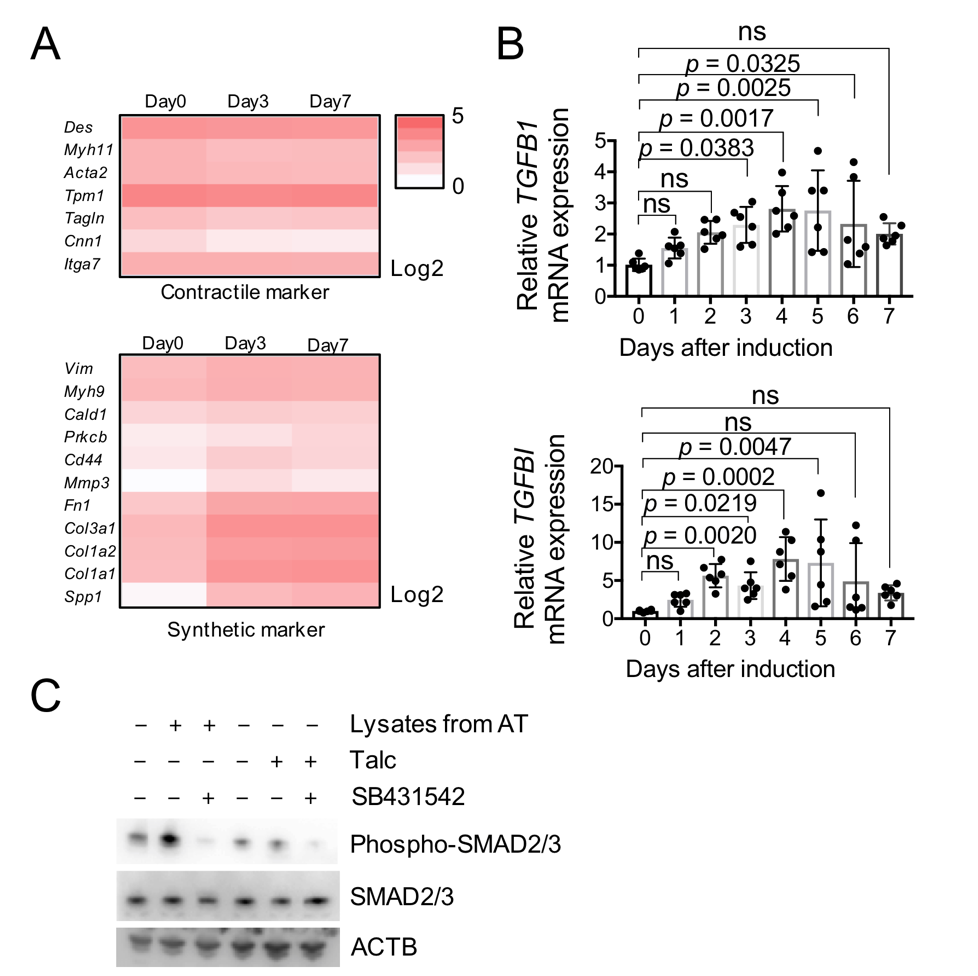
Supplemental Figure 8. Expression analysis of TGFB and marker genes of VSMCs with contractile and synthetic phenotypes.** A. Heatmaps illustrating mRNA expression levels on days 0, 3, and 7 for VSMCs with contractile (*upper panel*) and synthetic (*lower panel*) phenotypes in heart tissues based on next-generation sequencing data. B. Quantitative RT-PCR for *TGFB1* and *TGFB-induced* genes. Values are expressed as mean ± standard error (n=6 in each group). Statistical analysis for *TGFB1* and *TGFBI* was performed using one-way ANOVA with Dunnett's multiple comparison test and Kruskal–Wallis test with Dunn's multiple comparisons test (nonparametric). C. Adhesion tissue around the heart tissue was obtained from talc-injected mice (day 7) and homogenized with Dulbecco's Modified Eagle Medium (DMEM). After centrifugation, the supernatants were added into the MILE SVEN 1 cells (murine endothelial cells) cultured with serum-starved DMEM, followed by culturing for 1 hour. Then, the endothelial cells were co-cultured with or without 1 μM SB431542. Protein concentrations of cell lysates were measured by DC/RC Protein Assay Kit (Bio-Rad), and 20 μg of extracted proteins were used for western blotting analysis with anti-phosphorylated SMAD2 (Cell signaling., Phospho-Smad2 (Ser465/467) (138D4)., catalog no. 3108., 1:1000 dilution), anti-SMAD2/3 (D7G7) (Cell signaling., catalog no. 8685), or Anti-β-Actin antibody (catalog no. A5441). Chemiluminescent images were obtained using LAS4000.

**
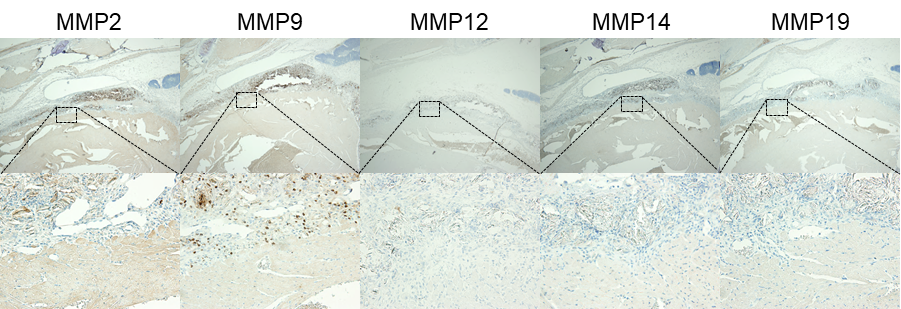
**

**Supplemental Figure 9. Tissue distribution of MMP2, MMP9, MMP12, MMP14, and MMP19 in mice with pericardial adhesions.** The chest cavities were excised from mice on day 7 after talc injection, and immunohistochemical staining with anti-MMP2 (1:1000), anti-MMP9 (1:1000), anti-MMP12 (1:1000), anti-MMP14 (1:1000), and anti-MMP19 (1:1000) antibodies was conducted. Scale bar = 20 μm. Antibodies used in this study are listed in Supplementary Table 2.


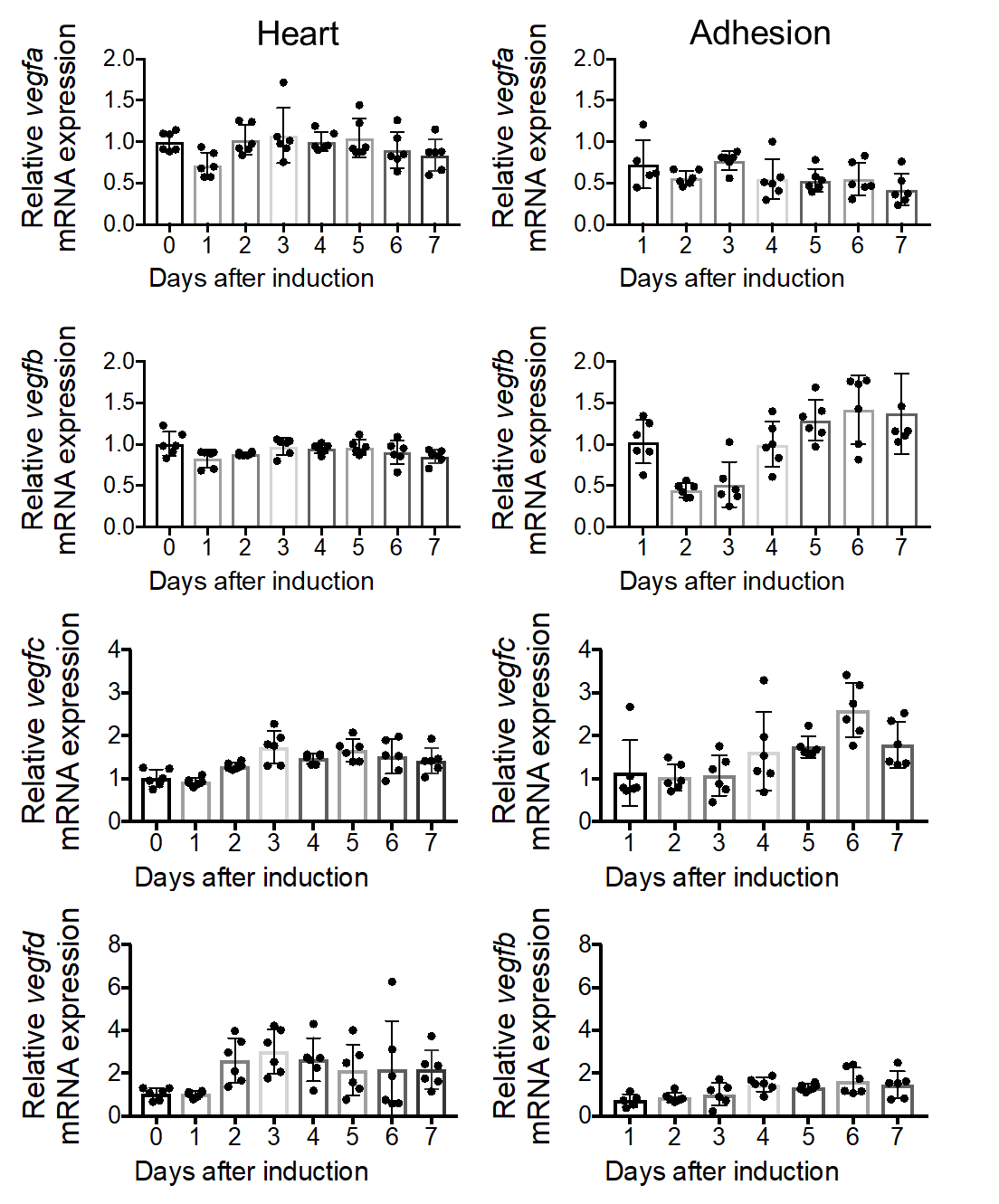


**Supplemental Figure 10. Quantification of mRNA for *vegf* family members in adhesion and heart tissues.** mRNA levels of *vegfa, vegfb, vegfc*, and *vegfd* in heart and adhesion tissues were quantified by quantitative RT-PCR analysis. Values are expressed as mean ± standard error (n = 6 in each group). Primers used in this study are listed in Supplementary Table 1.

**Supplementary Tables**

**Supplementary Table 1. Primer sequences for quantitative RT-PCR in Supplemental Figures 5, 8, and 10.**

| **Target gene** |  | **Primer sequence (5′-3′)** |
| --- | --- | --- |
| *COL1A1* | forward | CCCTGGTCCTCGAGGTCGCA |
|  | reverse | TTCTTGCGGCTGCCTTCGGG |
| *COL1A2* | forward | GCAGGTTCACCTACTCTGTCCT |
|  | reverse | CTTGCCCCATTCATTTGTCT |
| *FN1* | forward | CGAGGTGACAGAGACCACAA |
|  | reverse | CTGGAGTCAAGCCAGACACA |
| *MMP9* | forward | CGAACTTCGACACTGACAAGAAGT |
|  | reverse | GCACGCTGGAATGATCTAAGC |
| *TGFB1* | forward | TTGCTTCAGCTCCACAGAGA |
|  | reverse | TGGTTGTAGAGGGCAAGGACC |
| *TGFBI* | forward | CGCCAAGTCACCCTACCAG |
|  | reverse | TGCACAGCACATACATTGGGG |
| *vegfa* | forward | TTTACTGCTGTACCTCCACCA |
|  | reverse | ATCTCTCCTATGTGCTGGCTTT |
| *vegfb* | forward | CCTGGAAGAACACAGCCAAT |
|  | reverse | GGAGTGGGATGGATGATGTC |
| *vegfc* | forward | GGGAAGAAGTTCCACCATCA |
|  | reverse | ATGTGGCCTTTTCCAATACG |
| *vegfd* | forward | GCTGTCACTGTTGCCCACTA |
|  | reverse | CCCTTCCTTTCTGAGTGCTG |
| *GAPDH* | forward | ATGTAGGCCATGAGGTCCAC |
|  | reverse | TGCGACTTCAACAGCAACTC |

**Supplementary Table 2. Antibodies used in the experiments for Supplementary Figure 9**

| **Target antigen** | **Source** | **Catalog #** |
| --- | --- | --- |
| *MMP2* | Abcam | ab97779 |
| *MMP9* | Abcam | ab38898 |
| *MMP12* | Abcam | ab231096 |
| *MMP14* | Abcam | ab53712 |
| *MMP19* | Abcam | ab216633 |
